# The effect of cryoprotectant and storage conditions on the aggregation of poly(ethylene glycol)-poly(α-benzyl carboxylate-ε-caprolactone) nanoparticles

**DOI:** 10.3389/jpps.2026.15721

**Published:** 2026-02-13

**Authors:** Nasim Sarrami, Soheyla Honary, Mohammad Reza Vakili, Afsaneh Lavasanifar

**Affiliations:** 1 Faculty of Pharmacy and Pharmaceutical Sciences, University of Alberta, Edmonton, AB, Canada; 2 Department of Chemical and Materials Engineering, Faculty of Engineering, University of Alberta, Edmonton, AB, Canada

**Keywords:** cryoprotectant, freeze-thaw, nanoparticles, PEG, storage conditions

## Abstract

**Introduction:**

The preparation of nanoparticles (NPs) in aqueous media leads to thermodynamic instability and aggregation during storage. The objective of this study was to identify an optimum condition for the storage of poly(ethylene glycol)-poly(α-benzyl carboxylate-ε-caprolactone) (PEG-PBCL) NPs with minimum to no NP aggregation.

**Methods:**

Nanoparticles were prepared from PEG-PBCL of varied PBCL degrees of polymerization. Prepared NPs were either subjected to freeze-thaw or lyophilization with the addition of sugars or polyethylene glycols (PEGs) of varying molecular weight. The average diameter and polydispersity of NPs before and after freeze-thaw or lyophilization/reconstitution in water was assessed by dynamic light scattering and transmission electron microscopy (TEM). Differential Scanning Calorimetry (DSC) was used to compare the thermal behaviour of NPs before and after selected storage conditions.

**Results:**

Irrespective of the DP of PBCL, minimum size growth in the freeze-drying method was achieved when PEG3350 and methoxy-PEG 5000 were used as cryoprotectant at a w/w ratio of 4:1 to PEG-PBCL. Under this condition, NP size increased about 2-fold after reconstitution. In the freeze-thaw method, both sucrose and PEGs of different molecular weights, protected the PEG-PBCL NPs of different PBCL length from significant size growth where average particle size growth was not more than 1.20 folds.

**Conclusion:**

Our findings suggest that freeze-thawing of PEG-PBCL NP using sucrose or PEG can prevent the NP aggregation and is the best method for PEG-PBCL NP storage.

## Introduction

Nanoparticles (NPs) are nanoscale engineered materials characterized by at least one external dimension within the range of 1–100 nm with the potential to alter the bioavailability of compounds encapsulated within them [1]. Owing to these unique properties, NPs have increasingly attracted attention as promising vectors for drug and gene delivery in the pharmaceutical industry [2]. NPs act as vehicles that can improve the solubility of hydrophobic compounds, control drug release, bypass biological barriers against drug delivery and/or preferentially deliver their cargo to the site of drug action in the biological system [3, 4]. These characteristics may eventually lead to an increase in the therapeutic index of many medications or drug candidates [3].

The U.S. Food and Drug Administration (FDA) requires that each premarket product report characteristics such as chemical composition, particle size and size distribution, particle shape and morphology, and physicochemical stability [1].

For biological use, NPs are commonly prepared in aqueous media. Under this conditions, NPs are generally thermodynamically unstable and prone to aggregation upon storage [5]. For pharmaceutical use, NPs are required to retain their initial physicochemical properties and quality in terms of chemical structure integrity, average diameter, polydispersity, morphology and drug encapsulation as well as release properties throughout their shelf life [6]. A rise in temperature, can affect the stability of NPs with regards to one or a few of the above properties. For this reason, NP products are usually stored in cold temperatures or in a frozen state. This has been the case for COVID-19 mRNA lipid nanoparticle (LNP) based vaccines which require storage at −20 or −80 °C [7]. However, the requirement for keeping the NPs at such low temperatures, imposes limitations in their distribution and storage in remote locations that do not have access to devices with controlled freezing temperatures. An alternative solution is the removal of water from the prepared NPs and NP reconstitution at the time of use, which can not only facilitate the distribution and use of these products, but also extend their shelf life.

In order to remove water from the formulations, freeze-drying also known as lyophilization is a popular method that can make NPs amendable for storage at room temperature (RT) [6, 8]. Freeze drying is comprised of three main steps of freezing, primary evaporation and secondary evaporation of water [9]. Briefly, after the sample is frozen, sublimation of frozen water accrues during the primary evaporation followed by desorption of the remaining water during the secondary evaporation [6, 8–10]. All three freeze-drying steps cause stress on NPs [10]. Among these steps, the freezing step causes the most stress that can lead to aggregation and growth in NP average diameter as well as polydispersity [11, 12]. Freeze-drying of NPs is considered successful when the physicochemical properties of the NPs, such as average particle size, polydispersity and drug encapsulation, are preserved following reconstitution. The dried product is also expected to show easy and rapid reconstitution [13, 14].

To overcome the stresses caused by freezing and/or freeze-drying, addition of cryoprotectants to NP solutions are commonly required [13]. Different compounds such as sugars (sucrose, mannose, trehalose, etc.) [15], or hydrophilic polymers (poly(ethylene glycol) (PEG) [5], polyvinylpyrrolidone (PVP) [16], etc.) have been reported to function as cryoprotectants. In general, the higher concentrations of cryoprotectants, and the more rapid freezing is expected to lead to a better NP re-dispersion [10].

Each NP formulation requires its unique conditions for lyophilization since this process is dependent on the nature of the particles and their chemical structure [5]. Most reports in the literature have focused on the lyophilization of liposomes and solid lipid NPs. Reports on freeze-drying of polymeric NPs are limited [11, 12, 17]. The focus of the present study was to investigate proper conditions for the storage of poly(ethylene oxide)-*block*-poly(α-benzyl carboxylate-ε-caprolactone) (PEG-PBCL) NPs through lyophilization or freeze-thaw methods. In this context, the effect of different cryoprotectants, their concentration, freezing and storage conditions on the physicochemical properties of reconstituted or thawed PEG-PBCL NPs was assessed.

PEG-PBCL is a biocompatible and biodegradable block copolymer that has been used to improve the water solubility of poorly soluble drugs and/or enhance the delivery of anticancer agents to solid tumors [18–20]. Accordingly, the effect of storage conditions on the physicochemical properties of drug loaded PEG-PBCL NPs is of interest. In this manuscript, in addition to blank PEG-PBCL NPs, we investigated the effect of storage condition on the properties of PEG-PBCL NP formulations of two model investigational drugs, namely A83B4C63 and A4. The two selected investigational drugs used in this study, have shown chemo and radio-sensitizing activity in cancer cells. A83B4C63 is a novel poorly water-soluble compound that inhibits a DNA repair enzyme known as polynucleotide kinase-phosphatase (PNKP). A4 is a novel inhibitor of another DNA repair enzyme known as ERCC1-XPF heterodimer. Development of PEG-PBCL NP formulations of A83B4C63 and A4 have been reported by our team previously [20–22]. Both A83B4C63 and A4 are poorly soluble drug candidates that have shown have shown acceptable encapsulation efficiency and release profiles as part of PEG-PBCL nanoparticle formulation in previous studies from our group.

The physicochemical properties of nanoparticles play an important role on their proper storage conditions and the type of cryoprotectant that can preserve the original characteristics of nanoparticles. Therefore, the results obtained in this manuscript cannot be naturally extended to other types of polymer-based nanoparticles. Similar reports from the literature on specific needs for the preservation of different nanoparticle structures attest to this conclusion [12, 23].

## Materials and methods

### Materials

Poly(ethylene glycol) (PEG) average molecular weight (Mn) of 1,450, 3,350, and 8,000 Da, methoxy PEG with average molecular weight of 2000 and 5,000 Da, sucrose and all solvents were purchased from Sigma (St. Louis, USA). α-Benzyl carboxylate-ε-caprolactone (BCL) monomer was prepared by Alberta Research Chemicals Inc. (Edmonton, Canada) based on previously reported methods [24]. Stannous octoate was purchased from Sigma (St. Louis, USA.) and purified in our lab. A83B4C63 and A4 were synthesized by the laboratory of Dr Fredrick West based on protocols reported in previous publications [21, 25]. Sucrose, sorbitol, lactose, and mannitol were purchased from Merck, Germany.

### Polymer synthesis

Block copolymers were synthesized based on previously reported studies through ring opening polymerization of BCL by PEG [21, 24, 26]. Briefly, methoxy PEG (5,000 Da) and BCL (different ratios to PEG depending on the intended DPs for PBCL block) were weighed and transferred to an ampule. This was followed by the addition of stannous octoate as catalyst. Afterward, the ampule was connected to a vacuum line and sealed. The ampule was placed in a 140 °C oven for 4 h. To purify the synthesized polymer, reaction contents were dissolved in dichloromethane and added to the excess amount of hexane to precipitate the synthesized polymer. The mixture was decanted to separate the solid product from supernatant. The polymer was left under vacuum so that the residual solvents were evaporated. The synthesized polymers were characterized using a 600-MHz Bruker NMR instrument (Bruker Instruments, Inc., Billerica, MA, USA). Polymers were dissolved in deuterated chloroform, CDCl_3_ for ^1^H NMR analysis. The ratio of the peak intensity of the methylene hydrogens of PEG (-C**H**_2_C**H**_2_O-, δ = 3.65 ppm) to the methylene hydrogens of PBCL backbone (-OC**H**_2_-, δ = 4.1 ppm) was used to calculate the average molecular weight (Mn) of PBCL block assuming a 5,000 Da molecular weight for PEG [24, 27].

#### Nanoparticle preparation

PEG-PBCL NPs were prepared based on previous works [24, 28]. In brief, 10 mg of the polymer was weighed and dissolved in 0.25 mL acetone and added dropwise to 5 mL deionized water (DiH_2_O) while stirring. The sample was left stirring overnight to evaporate the acetone. After acetone evaporation, the NP size and polydispersity were measured using dynamic light scattering (DLS) (either Nano-ZS90, or Ultra, Zeta-Sizer, Malvern Instruments Ltd, UK were used). All samples were measured at 2 mg/mL polymer concentration at 25 °C with a 173° angle scattering unless mentioned otherwise.

For the preparation of A83B4C63 loaded PEG-PBCL NPs a previously developed method was used [20, 21]. Briefly, 1 mg of A83B4C63 and 10 mg of PEG-PBCL_22_ polymer were weighed and dissolved in 0.25 mL acetone and added dropwise to 5 mL of DiH_2_O under stirring. Then the sample was left overnight for the acetone to evaporate. After acetone evaporation, the sample was centrifuged at 10,000 x g for 10 min to spin down the un-encapsulated A83B4C63. To determine encapsulation efficiency, a 20 µL sample of the purified A83B4C63 loaded NP was taken and 80 µL dimethyl sulfoxide (DMSO) was added, followed by measuring the UV absorbance of A83B4C63 at 400 nm using a plate reader (BioTekInstruments Inc.).

PEG-PBCL NPs encapsulating A4 were developed based on previous work [22, 29]. In a typical experiment, 0.5 mg of A4 and 10 mg of PEG-PBCL_22_ were weighed and dissolved in 0.25 mL acetone followed by dropwise addition to 5 mL DiH_2_O under stirring. The sample vial was left uncapped under fume hood to evaporate acetone overnight. To purify the sample from un-encapsulated A4, the prepared sample was centrifuged at 10,000 x g for 10 min. To calculate the encapsulation efficiency of A4, 80 µL DMSO was added to a 20 µL of the NP dispersion. UV absorption of the drug was measured using a plate reader (BioTekInstruments Inc.) at 470 nm.

The encapsulation efficiency was calculated using the following equation:

$$\text{EE }\%=\frac{\text{the}\;\text{amount}\;\text{of}\;\text{encapsulated}\;\text{drug}\;}{\text{the}\;\text{total}\;\text{amount}\;\text{of}\;\text{drug}}X100$$

### Freeze-drying method

#### Selection of the best sugar as cryoprotectant

Nanoparticle dispersions in DiH_2_O were placed in Eppendorf tubes (Eppendorf, USA) and rapidly frozen in a mixture of dried ice and acetone. The freeze-drying process was carried out by VirTis Lyo-Centre lyophilizer, USA. The freeze-dried sample was reconstituted with 1 mL of DiH_2_O with manual shaking. The resulting colloidal dispersion was then used for particle size analysis by DLS (MALVERN Nano-ZS90 ZETA-SIZER, Malvern Instruments Ltd, Malvern, UK). Different sugars (sucrose, mannitol, trehalose, lactose, and sorbitol) were first evaluated to find the appropriate cytoprotectant for the storage of PEG-PBCL NPs dried using freeze-drying method. The concentration of sugars was 5 mg/mL, i.e. 14.6 mM of sucrose, 27.4 mM mannitol, 13.2 mM of trehalose, 14.6 mM lactose and 27.4 mM sorbitol. Sugars were added to PEG-PBCL NPs in a 1:10 w/w ratio.

In the present study, a 11 trial, 2-factor, 3-level Box-Behnken factorial design was used to study the optimal experimental conditions for freeze-drying PEG-PBCL_14_ NPs when using sucrose as the cryo-protectant [30].

#### Assessing the effect of PEG versus sucrose as cytoprotectant

The PEG-PBCL NPs (2 mg/mL) were divided into 1 mL aliquots and transferred to 2 mL Eppendorf tubes (Eppendorf, USA). Appropriate volumes of PEG, methoxy PEG or sucrose from stock solution were added to each aliquot. The samples with or without cryoprotectants were completely frozen at −80 °C freezer. Frozen samples were transferred to the freeze-dryer chamber (Labconco, USA) with the collector set to −85 °C and vacuum set to 0 mbar for 48 h. Freeze-dried samples were reconstituted in 1 mL of DiH_2_O with moderate shaking. Nanoparticle average diameter and PDI were measured using DLS as described above.

### Freeze-thaw method

The PEG-PBCL NPs (2 mg/mL) were divided into 1 mL samples and frozen by either snap-freezing via liquid nitrogen or placed in a −20 or −80 °C freezer. After samples were completely frozen for 4–5 h, they were transferred to a heat block (VWR digital heat block, VWR, USA) set to 25 °C until thawed. Nanoparticles’ size and PDI were measured by DLS.

To assess the role of cryoprotectants, 1 mL of PEG-PBCL NPs (2 mg/mL) with or without cryoprotectants were subjected to freezing at −80 °C freezer and thawed on a heat block (VWR digital heat block, VWR, USA) set to 25 °C. Nanoparticles’ size and PDI were measured using DLS as described above. The added cryprotectants included sucrose, PEGs with molecular weights of 1,450, 3,350, and 8,000 Da and methoxy PEG with molecular weights of 2000 and 5,000 Da. The w/w ratio of PEG to the PEG-PBCL polymer of 0.25:1, 0.50:1, 1:1, 2.00:1 and 4.00:1 were used. The w/w ratio of sucrose to the PEG-PBCL polymer was 13.25:1.

### Response measurement

The Z average diameters before and after the freeze-thawing and freeze-drying processes of samples without or with different concentrations of the cryoprotectant were measured by DLS as described above. The response for each sample was calculated as follows:

Response=Sf/Si

In the presented article, Si refers to the initial size of the NPs describing the size of the NPs before storage while Sf refers to the final size of NPs after storage using either freeze-drying or freeze-thawing methods of storage. The size ratio was used as a response (dependent variable) for the optimization study.

### Transmission electron microscopy (TEM)

The morphology of PEG-PBCL NPs with or without methoxy-PEG5000 as cryoprotectant before and after freeze-drying was determined by TEM (Morgagni TEM, Field Emission Inc., USA). In brief, a drop of NP solution with a polymer concentration of 2 mg/mL was spotted on a copper-coated grid. The grid was kept horizontal for 1 min allowing the particles to settle. Afterward, the excess sample was removed from the grid. The grid was negatively stained with 2% phosphotungstic acid and the excess removed after 2 min. Then the grid was placed into the TEM for visualization.

### Differential scanning calorimetry (DSC)

The thermal behavior of PEG-PBCL block copolymers and PEG-PBCL NPs with PBCL degree of polymerization of 22 after freeze-drying with and without PEG were assessed by DSC (TA instrument, USA). The samples were weighed (1–5 mg) and sealed in Tzero aluminum pans. The heating rate was 10 °C/min starting from 25 to 120 °C under a nitrogen atmosphere. An empty Tzero pan was set as the reference.

### Statistical analysis

The experimental design and regression analysis were carried out by either SPSS 22 or Prism 8.0 as identified for each experiment in the results section. The significance of independent variables and their interactions were tested by t-test, one-way and two-way analysis of variance (ANOVA). P value of 0.05 was used to determine the statistical significance. Various statistical indices such as t value, p value, F value, correlation coefficient (R), determination coefficient (R^2^), and adjusted determination coefficient (adj R^2^), were used to assess the statistical significance of the quadratic models. Composite desirability values were used for comparison of different fabrication conditions.

## Results

### Characterization of synthesized polymers

^1^H NMR spectra interpretations were reported in our previous publications [24]. Comparison of the peak intensity of PEG (-CH_2_CH_2_O-, δ 3.65 ppm) to that of PBCL (-OCH_2_-, δ 4.1 ppm) showed the preparation of PEG-PBCL polymers with a DP of 9, 14 or 22 for the PBCL block from the polymerization procedures. The DP of polymers is shown as a subscript for polymers under study in the paper.

### Selection of the best sugar as cryoprotectant for the freeze-drying of PEG-PBCL_14_ NPs

In a preliminary study, different sugars were tested to find the appropriate cryoprotectant for the storage of PEG-PBCL NPs. Table 1 shows the results for 6 different sugars under study. The concentration of NPs and sugars was chosen according to previous literature and pilot studies [8, 24]. Table 3 compares the effect of adding different sugars on the average diameter of NPs before and after freeze-drying and reconstitution. The results show addition of sucrose as cryoprotectant lead to the least increase in the average size of NPs reflecting minimum NP aggregation. Therefore, sucrose was chosen as the sugar cryoprotectant for studies in the next steps.

**TABLE 1:** Z average diameter of PEG-PBCL_14_ NPs before addition of cryoprotectant (Si) and after (Sf) the freeze-drying process and the ratio of Sf/Si using different sugar as cryoprotectant.

Name of sugar	Si (nm) N = 2	Sf (nm) N = 2	Ratio Sf/Si
Sucrose	46.3 ± 1.8	81.36 ± 2.1	1.8
Mannitol	47.3 ± 2.2	92.24 ± 3.4	2
Trehalose	48.5 ± 2.3	654.5 ± 5.4	13.5
Lactose	46.7 ± 1.9	117.2 ± 6.3	2.5
Sorbitol	46.1 ± 1.9	178.8 ± 7.1	3.9

In the following step, the effect of two different factors, i.e., copolymer and sucrose concentration on the average diameter of NPs before and after freeze-drying and reconstitution was assessed. Three different concentrations of PEG-PBCL and sucrose (Table 2) were studied. Table 3 summarizes the results of this study which was performed using a central composite design.

**TABLE 2:** The two factors and the corresponding three-level settings for lyophilization of PEG-PBCL_14_ NPs: X1(sucrose concentration); X2 (copolymer concentration).

Level	X1 (mg/mL)	X2 (mg/mL)
−1	3	1
0	26.5	2
+1	50	3

**TABLE 3:** Experimental conditions for central composite design and Z average diameter of PEG-PBCL_14_ NPs before the addition of cryo-protectant (Si) and after (Sf) freeze-drying process and the ratio of Sf/Si (response) using different concentration of sucrose as cryo-protectant.

Trial	X1	X2	Si (nm) N = 3	Sf (nm) N = 3	Response (Sf/Si)
1	+	+	23.57 ± 1.3	86.03 ± 1.9	3.62
2	+	-	23.08 ± 1.1	49.51 ± 1.7	2.15
3	-	+	20.13 ± 1.4	9,005 ± 5.2	447.34
4	-	-	19.85 ± 1.2	1585 ± 4.4	49.95
5	+	0	22.68 ± 1.3	44.74 ± 1.5	1.97
6	-	0	18.49 ± 1.1	3,511 ± 4.5	189.89
7	0	+	20.22 ± 1.2	42.07 ± 1.2	2.08
8	0	-	20.72 ± 1.2	44.77 ± 1.1	2.16
9	0	0	19.86 ± 1.3	57.39 ± 1.6	2.86
10	0	0	20.32 ± 1.2	50.50 ± 1.4	2.49
11	0	0	20.24 ± 1.3	55.86 ± 1.4	2.76

By applying the multiple regression analysis to the output data, the following second-order polynomial equation in coded form was established to explain the relationship between the dependent and the independent variables for PEG-PBCL_14_ NPs, during the freeze-drying (Equation 1).

$$Y=2.470-113.240\;X1+66.463\;X2+113.350\;X1^{2}-98.980\;X1X2$$ (1)

Where Y1 is the Sf/Si ratio, X1 and X2 are sucrose concentration and copolymer concentration, respectively. Experiments were performed in a random order on three levels for each factor using Box-Behnken design. In Equation 1, the interaction term (X1X2) shows how the response changes when two variables are simultaneously changed, while the effect of changes in each single variable on the response is reflected by the main effect terms (X1and X2) and quadratic term (X1^2^). As it could be observed, the second-order of the independent variable X2 was not included in the equation, due to its minimal contribution to the response.

The analysis of variance (ANOVA) was conducted to test the significance and the lack of fit of the quadratic models for the experimental data. Fisher’s F-test for model is 17.116 and P = 0.002. This indicates that the model is able to explain a significant amount of variation on the dependent variable (Sf/Si). The level of the fit for the model was checked by the determination coefficients (R = 0.959 and R^2^ = 0.919), which indicates that up to 92% of the response can be explained by this model. Moreover, the high value of the correlation coefficient demonstrates a good correlation between the observed responses and the responses predicted by the model. The value of the adjusted coefficient of determination (adj R^2^) was also high for the model (adj R^2^ = 0.866), which confirms the high significance of the model.

The significance of different model components (Equation 1) was determined by Students’t-test, the results of which are demonstrated in Supplementary Table S1. The t- value, for each component is a measure of that parameter’s standardized effect. The larger the magnitude of t-value, the more significant is the corresponding parameter in the model. The most significant component of the quadratic model was the sucrose concentration, which showed the highest value of t, among all components (t = −5.445; P = 0.002). Besides, a negative correlation between sucrose concentration (X1) and response was observed. The interaction term (X1X2) was the second most influential component (t = 3.886; P = 0.008). Polymer concentration (X2) also influenced the average diameter of micelles following a positive correlation (t = 3.196; P = 0.019). However, no significant effect on response was observed for X2^2^.

Based on this model, the responses for more than 337 conditions for PEG-PBCL_14_ NPs were predicted. The criteria of design points were determined based on pretests performed in our lab considering the feasibility of each condition. More than 15 optimum conditions were identified, and used for the preparation of NPs, in order to compare the predicted and experimental response. Then, the correlation between the model predictions and the observed response can be visualized by the parity plots (Supplementary Figure S1A), which demonstrate a satisfactory correlation between the predicted and observed values and small deviations between the experimental and predicted responses. The results indicate the good fit of the model. The residual plot (Supplementary Figure S1B), shows random distribution of residuals without any trend, indicating good prediction of the maximum response along with constant variance. The data further confirmed the adequacy of the quadratic models in describing the most important conditions influencing the size of prepared micelles during the self-assembly process.

In general, the data showed that a sucrose concentration of ≥26.5 mg/mL was needed for PEG-PBCL_14_ NPs at 2–3 mg/mL to show ∼ 2-fold aggregation upon freeze-drying and reconstitution. A concentration of 3 mg/mL of sucrose was not adequate to prevent the aggregation of PEG-PBCL_14_ NPs upon freeze-drying and reconstitution.

### Freeze-drying of PEG-PBCL_22_ NPs using PEG or sucrose as cryoprotectant

**FIGURE 1:**
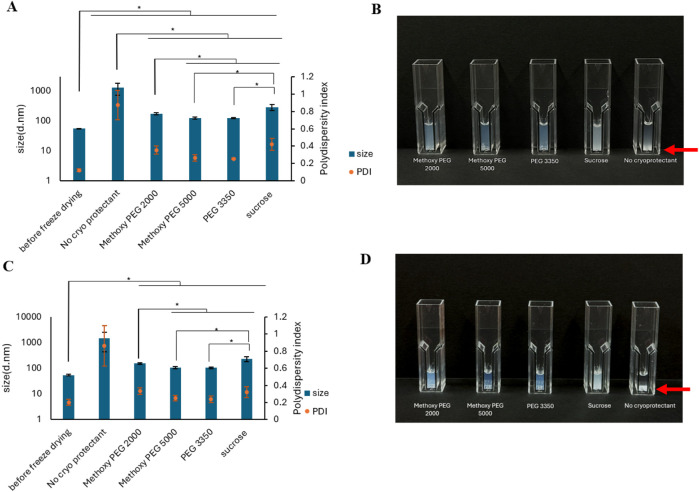
**(A,C)** Average diameter and PDI after reconstitution of freeze-dried PEG-PBCL_22_ NPs using different cryoprotectants in 1 mL of DiH_2_O compared to NP size and PDI before freeze drying. **(B,D)** Visual appearance of reconstituted PEG-PBCL_22_ NPs with different cryoprotectants. The w/w ratio of PEG: and sucrose to PEG-PBCL_22_ was 2:1 and 13.25:1, respectively in Figures **A** and **B** (* represent p < 0.05, One-way ANOVA, n = 3). The w/w ratio of PEG: and sucrose to PEG-PBCL_22_ was 4:1 and 13.25:1, respectively, in Figures **C** and **D**. (* represent p < 0.05, One-way ANOVA, n = 3). All samples were frozen at −80 °C followed by drying process. Red arrow indicates the precipitation of PEG-PBCL_22_ NPs.

In the next step we used PEGs of different molecular weight and end group as cryoprotectant applied at 1:2 or 1:4 PEG: PG-PBCL ratio to avoid PEG-PBCL NP aggregation during freeze-drying. Based on the data obtained on PEG-PBCL_9_ NPs (Supplementary Figures S2, S3; Supplementary Tables S2–S4), we have selected methoxy PEG 2000, methoxy PEG 5000 and PEG 3350 at w/w ratios of 1:2 and 1:4 to PEG-PBCL_22_ to study the effect of cryoprotectant and its concentration on the aggregation of PEG-PBCL NPs of higher hydrophobic chain length. Sucrose with the w/w ratio of 13.25:1 to PEG-PBCL_22_ was also used as another cryoprotectant for these NPs. The samples without cryoprotectant showed formation of sediments when left for 2–3 min. The supernatant from these samples showed formation of aggregates that were 23.09-fold larger than NPs before freeze-drying (Figure 1). Adding sucrose led to a 4 to ∼ 5- fold increase in the average diameter of PEG-PBCL_22_ NP after freeze-drying and reconstitution compared to before freeze-drying (Table 4). On the other hand, the increase in size between after and before freeze-drying and reconstitution when PEGs were used as cryoprotectants was 2- to 3-folds on average (Table 4).

**TABLE 4:** The effect of cryoprotectant on the PEG-PBCL_22_ NP average size growth (Sf/Si) in freeze-drying and reconstitution procedure.

Cryoprotectant	Cryoprotectant: PEG-PBCL_22_ ratio (w/w)	Response (Sf/Si)
No cryoprotectant	0.0:1	24.88
Methoxy PEG 2000	2.0:1	3.14
Methoxy PEG 5000		2.27
PEG 3350		2.25
Methoxy PEG 2000	4.0:1	2.85
Methoxy PEG 5000		1.93
PEG 3350		1.94
Sucrose	13.25: 1	4.71

In general, the increase in NPs’ size after freeze-drying/reconstitution compared to before freeze-drying was lower when a PEG:PEG-PBCL_22_ ratio of 4:1 was used and when the MW of PEG was raised. At the 4:1 ratio of PEG 3350 Da and methoxy PEG 5000 Da to PEG-PBCL_22_, there was 1.94 and 1.93 fold increase in the average diameter of NPs, respectively (Table 4). There was no significant difference in the measured PDI value for NPs before and after freeze-drying/reconstitution at this condition (p > 0.05, One-way ANOVA), pointing to the relative success of these two materials as cryoprotectants compared to others under study in the freeze-drying process for PEG-PBCL_22_ NPs (Supplementary Tables S5, S6).

#### The effect of PBCL MW on the aggregation of PEG-PBCL NPs upon freeze-drying and reconstitution

Without any cryoprotectant, both PEG-PBCL NPs under study showed a significant increase of their average diameter following freeze-drying and reconstitution (around 4.0- and 24.9- fold increase in Sf/Si for PEG-PBCL_9_ and PEG-PBCL_22_ NPs, respectively) (Table 4; Supplementary Tables S3, S4, S6). This points to less aggregation of PEG-PBCL NPs when PBCL chains are shorter. In line with this observation, upon addition of methoxy PEG2000 Da, methoxy PEG5000 Da and PEG 3350 Da at a weight ratio of 4:1 as cryoprotectant less aggregation was seen in PEG-PBCL_9_ NPs compared to PEG-PBCL_22_ NPs (Supplementary Tables S3, S4, S6).

#### The effect of freeze-drying on the morphology of PEG-PBCL NPs investigated by TEM

**FIGURE 2:**
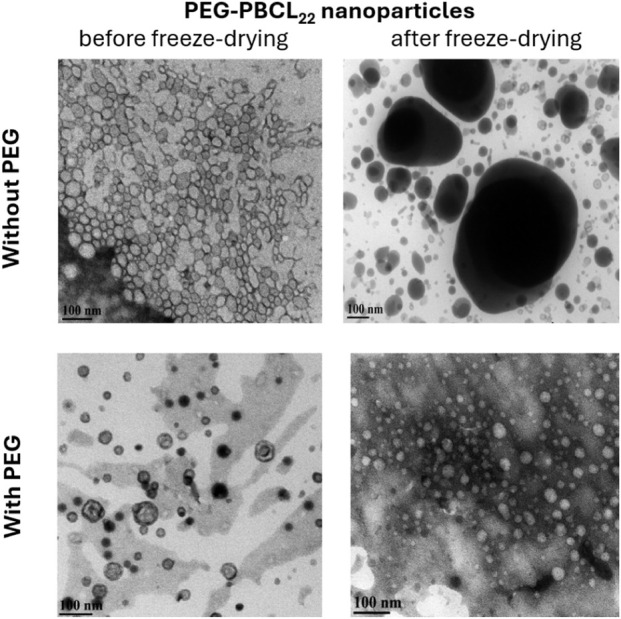
TEM images of PEG-PBCL_22_ NPs before and after freeze-drying with methoxy PEG (MW = 5,000 Da) with the w/w ratio of 4:1 of PEG: PEG-PBCL_22_. All samples were frozen at −80 °C followed by drying process reconstitution in 1 mL of DiH_2_O.

Figure 2 shows the TEM images of PEG-PBCL_22_ NPs of two different PBCL MWs before and after freeze-drying, with and without PEG. The NP sizes from TEM images seem to be inline with what was measured with DLS. Also, when PEG-PBCL NPs were freeze-dried without PEG, aggregation was seen. However, these aggregations were not seen when PEG was added as a cryoprotectant.

#### Freeze-drying of A83B4C63- and A4 loaded PEG-PBCL_22_ NPs

**FIGURE 3:**
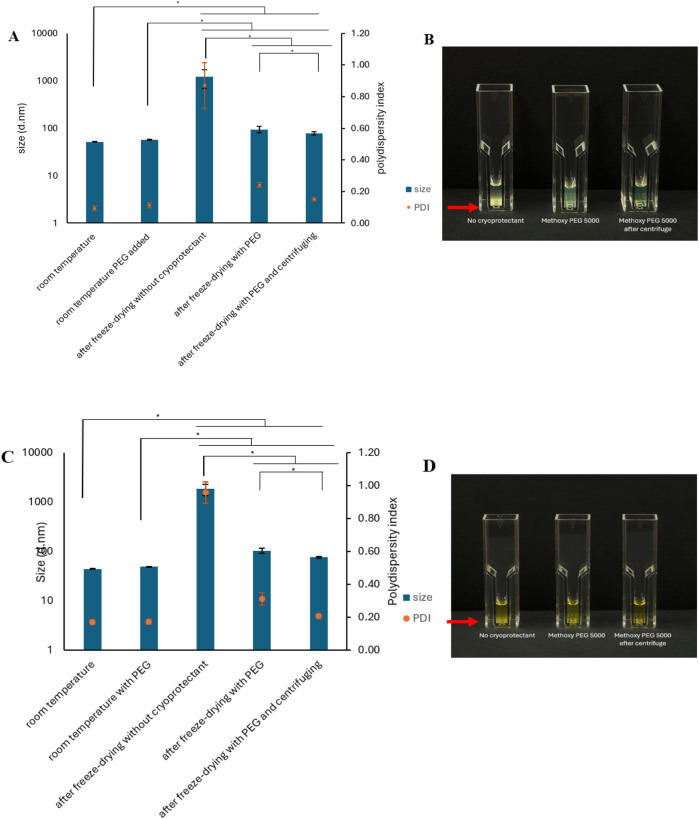
Average diameter and PDI after freeze-drying and redispersing PEG-PBCL_22_ NPs loaded with **(A)** A83B4C63 or **(C)** A4 in 1 mL of DiH_2_O. The w/w ratio of methoxy PEG (5,000 MWt): PEG-PBCL_22_ was 4:1 (* = p < 0.05, One-way ANOVA, n = 3). Visual appearance of reconstituted **(B)** A83B4C63 or **(D)** A4 loaded PEG-PBCL_22_ NPs with and without cryoprotectant. Red arrow indicates the precipitation of PEG-PBCL_22_ NPs. To freeze-dry samples, all samples were frozen at −80 °C followed by drying process.

PEG-PBCL NPs are suitable candidates for delivery of hydrophobic compounds due to their hydrophobic core. We studied, the impact of freeze-drying on the drug-loaded PEG-PBCL_22_ NPs while the cryoprotectant methoxy PEG (MW = 5,000 Da) with the w/w ratio of 4:1 of PEG: PEG-PBCL_22_ was used. Initially, the encapsulation efficiency of A83B4C63 into the PEG-PBCL_22_ NPs was 88.2 ± 1.4%, and the NPs’ size and PDI were 51.87 ± 87 nm and 0.09 ± 0.01, respectively (Figure 3). After freeze-drying and reconstitution in 1 mL DDH_2_O, the NPs’ size and PDI for the group with methoxy PEG 5000 as cryoprotectant were 95.09 ± 14.55 nm and 0.24 ± 0.02, respectively, showing a 1.83- and 2.56- fold increase compared to before freeze-drying, respectively. The NPs’ size and PDI following centrifugation was reduced to 78.4 ± 6.45 nm and 0.15 ± 0.01, respectively, significantly lower than the values measured before centrifugation (p < 0.05, One-way ANOVA) (Figures 3A,B) (Supplementary Table S7). The encapsulation efficiency after freeze-drying and centrifuging the samples was 78.3 ± 7.0%. The visual examination of the samples matched the findings from DLS. The samples without the cryoprotectent formed sediment after 2–3 min (Figure 3B), which was similar to our observation on the blank PEG-PBCL22 NPs (Figures 1B,D).

Another hydrophobic compound, namely A4, was encapsulated in PEG-PBCL_22_ NPs. The A4 encapsulation efficiency into PEG-PBCL_22_ NPs before freeze-drying was 79.02 ± 14.77%. The NPs’ size and PDI were 44.47 ± 0.61 nm and 0.17 ± 0.01, respectively. As shown in Figures 3C,D, the NPs’ size and PDI significantly increased in the group of NPs that had methoxy PEG 5000 Da as cryoprotectant to 102.90 ± 12.16 nm and 0.31 ± 0.04, respectively (p < 0.05, One-way ANOVA). After centrifugation of the reconstituted NPs, the encapsulation efficiency of A4 after freeze-drying was 77.91 ± 12.43% (p > 0.05, One-way ANOVA). The NPs’ size and PDI significantly decreased after centrifugation to 76.11 ± 4.18 nm and 0.21 ± 0.01 compared to 102.90 ± 12.16 nm and 0.31 ± 0.04 before centrifugation, respectively (Supplementary Table S8). The results from DLS were consistent with the visual examination of the samples. The cryoprotectant-free samples showed sediment formation after 2–3 min (Figure 3D). The observation was in line with our observation for blank PEG-PBCL_22_ NPs (Figures 1B,D). Moreover, the effect of cryoprotectant on the increase in average diameter (Sf/Si) of PEG-PBCL_22_ NPs loaded with A83B4C63 and A4 following freeze-drying was in line with results from blank PEG-PBCL_22_ NPs (Table 5).

**TABLE 5:** The effect of cryoprotectant on the increase in average diameter (Sf/Si) of PEG-PBCL_22_ NPs loaded with A83B4C63 and A4 following freeze-drying, reconstitution and centrifugation procedures. Comparisons are made with blank NPs. The initial size of NPs before freeze-drying in the absence of cryoprotectant (Si) was 51.87 ± 0.87 nm and 44.47 ± 0.61 nm for A83B4C63 and A4 loaded NPs, respectively.

Cryoprotectent	Loaded compound	Response (Sf/Si)	Encapsulation Efficiency (%)
None	A83B4C63	23.37	​
Methoxy PEG 5000 (before centrifuge)		1.83	92.2 ± 5.5
Methoxy PEG 5000 (after centrifuge)		1.51	78.3 ± 7.0
None	A4	41.62	​
Methoxy PEG 5000 (before centrifuge)		2.31	78.70 ± 12.47
Methoxy PEG 5000 (after centrifuge)		1.71	77.91 ± 12.43
None	None	26.70	​
Methoxy PEG 5000		1.93	​

### Differential Scanning Calorimetry (DSC)

**FIGURE 4:**
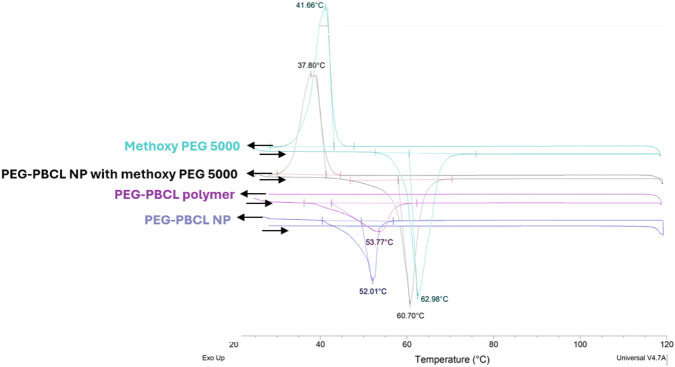
DSC thermograms for methoxy-PEG 5000, PEG-PBCL_22_ block copolymer, freeze-dried PEG-PBCL_22_ NPs, and freeze-dried PEG-PBCL_22_ NPs with methoxy PEG at a w/w ratio of 4:1 and PEG-PBCL_22_ polymers.

To assess the effect of freeze-drying and methoxy PEG addition as cryoprotectant on the thermal behaviour of PEG-PBCL_22_, DSC was performed. The DSC results as shown in Figure 4 indicated only one exothermic peak around 50 °C for PEG-PBCL_22_ or PEG-PBCL_22_ NPs alone. Methoxy PEG 5000 Da (alone) has shown a crystallization (∼41 °C) and a melting peak (∼62.98 °C). In PEG-PBCL plus PEG 5000 (added as a cryoprotectant at a 4:1 w/w ratio), these two peaks were also observed; at a slightly different position (37.8 and 60.7 °C). Interestingly the PEG as part of PEG-PBCL copolymer did not show the crystallization peak. Unlike free methoxy PEG, the PEG part of PEG-PBCL polymers did not crystalize in the presence of PBCL block.

### Freeze-thawing of PEG-PBCL NPs using PEG or sucrose as cryoprotectant

**FIGURE 5:**
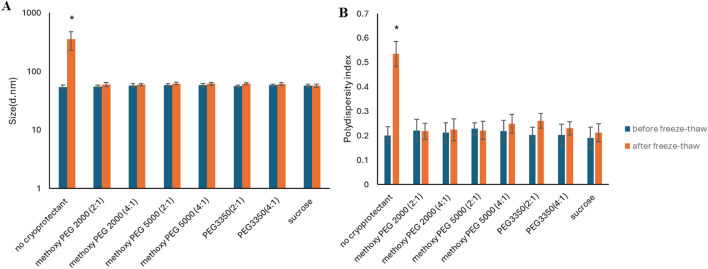
PEG-PBCL_22_ NPs’ **(A)** average diameter and **(B)** PDI before and after freeze-thawing without or with cryoprotectant. The (w/w) ratio of PEGs and sucrose to PEG-PBCl_22_ were 2:1, 4:1 and 13.25:1, respectively. (* represent p < 0.05, One-way ANOVA). All samples were frozen at −80 °C followed by thawing at RT.

The effect of freezing conditions (Supplementary Figure S4; Supplementary Table S9) and cryoprotectant (Supplementary Tables S10, S11) on the aggregation of PG-PBCL9 NPs was evaluated first. When PEG-PBCL_22_ NPs were subjected to the freeze-thaw procedure without the use of any cryoprotectant, the average size of NPs significantly enhanced by ∼ 6.5 fold (Table 6). In contrast, the NP size and PDI remained the same before and after freeze-thaw when a cryoprotectant was used (p > 0.05, One-way ANOVA). This effect was independent of the type and concentration of cryoprotectants under study (Figure 5) (Supplementary Tables S10–S12).

**TABLE 6:** The effect of cryoprotectant on the PEG-PBCL_22_ NP average size growth (Sf/Si) in the freeze-thaw procedure. The initial size of NPs before freeze-thawing in the absence of cryoprotectant (Si) was 54.5 ± 3.67 nm. All samples were frozen at −80 °C followed by thawing at RT.

Cryoprotectant	Cryoprotectant:PEG-PBCL_9_ ratio (w/w)	Response (Sf/Si)
None	0:1	6.49
Methoxy PEG 2000 before freeze-thaw Methoxy PEG 2000 after freeze-thaw	2:1	1.02 1.09
Methoxy PEG 5000 before freeze-thaw Methoxy PEG 5000 after freeze-thaw		1.07 1.14
PEG 3350 before freeze-thaw PEG 3350 after freeze-thaw		1.03 1.14
Methoxy PEG 2000 before freeze-thaw Methoxy PEG 2000 after freeze-thaw	4:1	1.06 1.09
Methoxy PEG 5000 before freeze-thaw Methoxy PEG 5000 after freeze-thaw		1.08 1.13
PEG 3350 before freeze-thaw PEG 3350 after freeze-thaw		1.07 1.11
Sucrose before freeze-thaw Sucrose after freeze-thaw	13.25: 1	1.05 1.05

#### The effect of PBCL MW on the aggregation of PEG-PBCL NPs upon freeze-thaw procedure

Freeze-thawing the PEG-PBCL NPs without cryoprotectants resulted in a growth in NPs diameter, irrespective of the PBCL MWt, but the fold increase in average dimeter of NPs was larger for PEG-PBCL_22_ NPs (fold increase in average dimeter upon freeze thaw was ∼1.8 and 6.5 for PEG-PBCL_9_ and PEG-PBCL_22_ NPs, respectively). However, once cryoprotectants were added, the size increase was independent of PBCL MW and the Sf/Si did not exceed 1.2 (Table 6), pointing to the success of this approach in preventing the aggregation of PEG-PBCL NP irrespective of PBCL MW.

## Discussion

Freeze-drying is a desired method to improve the shelf life of pharmaceutical products including NPs. Usually. Cryo-protectants need to be used before freeze-drying to prevent NP aggregation during the process. The optimum type and concentration of cryoprotectants should be identified for each NP structure [25, 26]. Here, we investigated the impact of different cryoprotectants on PEG-PBCL NPs stored using freeze-drying or freeze-thaw method in order to identify the best storage condition for these NPs.

The PEG-PBCL NPs were protected from freeze-drying stress using either sugar or polymers. The NPs’ size growth was inhibited by incorporating cryoprotectants (Figure 1; Supplementary Figures S2, S3) perhaps through formation of spatial obstacles that prevent NP aggregation [11]. After freeze-drying the PEG-PBCL_22_ NPs, the reconstitution of the particles without cryo-protectants led to the formation of suspensions showing precipitation (Figures 1B,D). This was not the case for any of the samples that had cryoprotectants, irrespective of the type and concentration. This observation was in line with other reported NPs that were freeze-dried without cryo-protectants [31, 32] and highlights the impact of cryo-protectants in the storage of PEG-PBCL NPs by freeze drying followed by reconstitution.

The freeze-drying process removes water molecules in samples including the ones present between the building blocks of the NPs. This process may affect the NPs structure in the dry state leading to NP aggregation and/or fusion. Sucrose molecules added to the sample, can replace water molecules present between the NPs and form hydrogen bonds with the PEG section of the PEG-PBCL NPs. This replacement and bond formation keeps the NPs structure intact through the loss of water in the freeze-drying process [8]. Besides, sucrose can form an amorphous glass upon freezing preventing NP aggregation by immobilizing NPs preventing NP aggregation.

However, PEG cryo-protectants showed better protection than sucrose for PEG-PBCL_22_ NPs against the freeze-drying stresses (Figure 1). When PEG is added as a cryo-protectant in NP samples, it can potentially insert itself to the NP structure providing not only a barrier against NP aggregation but also stealth properties avoiding exposure of NP cores to the media or other nearby NPs [33, 34]. PEG can also reduce the effect of particle crowding and buffer the osmotic pressure buildup preventing aggregation and fusion of particles during the freeze-drying process.

It has been reported that the addition of polymers such as PVA can stabilize and keep the NPs distant while they are going through the drying step of freeze-drying either by forming a matrix or coating the surface of the NPs [8, 35]. TEM images also showed that the NPs’ integrity was preserved when freeze-dried using PEG as cryo-protectant (Figure 2).

When the samples were studied using DSC, methoxy-PEG 5000 showed a crystallization and a melting peak (Figure 4). This was in line with previous reports [36, 37]. On the other hand, PEG-PBCL NPs and PEG-PBCL polymer, showed only one melting peak at around 52–53 °C indicating the interference of PBCL with the crystallization of PEG in the block copolymer and NP structure. PBCL is an amorphous polymer (due to the atactic structure of PBCL) that is not expected to show crystallization and melting [38]. Addition of PEG 5000 Da as cryo-protectant, led to the disappearance of PEG-PBCL melting peak pointing to the possible interference of PEG 5000Da in the interaction of PEG from different NPs keeping the PEG-PBCLs NPs distant from each other in the solid state (Figure 4).

We found that the stability of drug encapsulated PEG-PBCL NPs during freeze-drying process, depends on the encapsulated drug. Reconstitution of freeze-dried A83B4C63 loaded PEG-PBCL NPs led to a decrease in drug loading in NPs, but this did not occur for A4 loaded NPs (Table 5). This could be due to the leakage of A83B4C63 and/or precipitation of A83B4C63 NPs. In terms of NP size, after reconstitution of freeze-dried A83B4C63 NPs using methoxy PEG 5000 as cryoprotectant, 1.83- fold increase in NP size was observed. This increase for A4 loaded NPs was 2.31-fold (Figure 3).

Both PEG-PBCL_9_ and PEG-PBCl_22_ NPs have shown signs of aggregation even in the presence of different cryoprotectants when freeze-dried and reconstituted (Figure 1; Supplementary Figure S3; Table 4; Supplementary Tables S2–S6) and this method was less effective than the freeze-thaw method for the storage of NPs (Figure 5; Table 6; Supplementary Tables S7–S12).

Freezing conditions imposes the most stress on the NPs during freeze-thaw or freeze-drying procedures [8, 9]. The size and PDI of PEG-PBCL NPs showed an increase during freeze-thaw (Supplementary Figure S4), irrespective of the freezing method, due to an increase in NPs’ concentration in parts of the sample that are still in liquid state as the water media starts to form ice during freezing [10, 27]. However, the ratio of size increase was not similar in all employed freezing conditions. As a lower freezing temperature was used (i.e. freezing using liquid nitrogen or −80 °C freezer), the increase in size (or aggregation of NPs) was less in comparison to freezing at −20 °C freezer. This observation could be a result of forming a higher quantity and finer ice crystals at −80 °C compared to −20 °C [39]. Snap freezing using liquid nitrogen can also assist in this process, although we did not see any difference between the use of liquid nitrogen or storage at −80 °C freezer in the current study. Therefore, freezing NPs using the −80 °C freezer was considered the best approach, as it is more accessible than liquid nitrogen.

Overall, taking all data together, the best storage condition and cryoprotectant was found to be freezing of NPs at −80 C and thawing them using PEG 3350, methoxy PEG 2000, 5,000 Da or sucrose as cryoprotectants. Under this condition, minimum aggregation of NPs was seen and the original average diameter as well as PDI of NPs were preserved compared to before freezing. Although the increase in the average diameter of NPs in the absence of cryoprotectants was higher for PEG-PBCL_22_ NPs compared to PEG-PBCL_9_ NPs, addition of PEG 3350 Da, methoxy PEG 2000, 5,000 Da or sucrose as cryoprotectants was able to preserve the average size of both NPs upon freeze/thaw method of storage.

## Conclusion

The PEG-PBCL NP exhibited an increase in both the size and PDI upon freeze-drying. However, the use of cryo-protectants reduced the degree of NP aggregation. Among tested cryo-protectants, sucrose, PEG 3350 and methoxy-PEG 5000 Da exhibited superior control over NP size growth. Our studies suggest that the use of freeze-thaw method using sucrose, PEG of 3,350 and methoxy-PEG 2000 or 5,000 Da can preserve the original particle size and size distribution of PEG-PBCL NPs and is a better method for the storage of PEG-PBCL NPs irrespective of PBCL molecular weight compared to freeze-drying method. The presented findings provide an opportunity for further investigations on PEG-PBCL NPs properties when stored frozen for longer time periods (>1 week). Moreover, it would be worth while to assess the dissociation of PEG-PBCL NPs and PEG-PBCL degradation kinetics with and without cryoprotectant used here at various storage conditions, i.e. room temperature and 4 °C over time.

## Data Availability

The original contributions presented in the study are included in the article/Supplementary Material, further inquiries can be directed to the corresponding author.
