# Supplementary material for: The effect of cryoprotectant and storage conditions on the aggregation of poly(ethylene glycol)-poly(α-benzyl carboxylate-ε-caprolactone) nanoparticles
Source: J Pharm Pharm Sci. 2026 Feb 13;29:15721. doi: 10.3389/jpps.2026.15721 (PMC12945844; doi:10.3389/jpps.2026.15721)
Supplement: Supplementary file 1 [file Supplementaryfile1.pdf]

Supplementary Information

The effect of cryoprotectant and storage conditions on the aggregation of poly(ethylene glycol)-poly(α-benzyl carboxylate-ε-caprolactone) nanoparticles

Nasim Sarrami[1], Soheyla Honary[1], Mohammad Reza Vakili[1] and Afsaneh Lavasanifar[1, 2*]

[1]Faculty of Pharmacy and Pharmaceutical Sciences, University of Alberta, Edmonton, Alberta, Canada

[2] Department of Chemical and Materials Engineering, Faculty of Engineering, University of Alberta, Edmonton, Canada

*** Corresponding author**

Afsaneh Lavasanifar, PhD

Faculty of Pharmacy and Pharmaceutical Sciences, Department of Chemical and Materials Engineering, Faculty of Engineering, University of Alberta, Edmonton, Canada;

Phone: +1-780-492-2742;

afsaneh@ualberta.ca

Table S1. Coefficients for different components of the quadratic model for freeze-drying process

Variable	*Coefficients*	*Mean effect*	*t*	*P*
Intercept	***2.470***	***-***	***0.108***	***-***
X1	***-113.24***	***-0.631***	***-5.445***	***0.002***
X2	***66.463***	***0.370***	***3.196***	***0.019***
$X1^2$	***113.35***	***0.426***	***3.675***	***0.010***
X1 X2	***-98.98***	***-0.450***	***-3.886***	***0.008***

F=17.116; P=0.002; R=0.959; R^2=0.919; adj R^2= 0.866

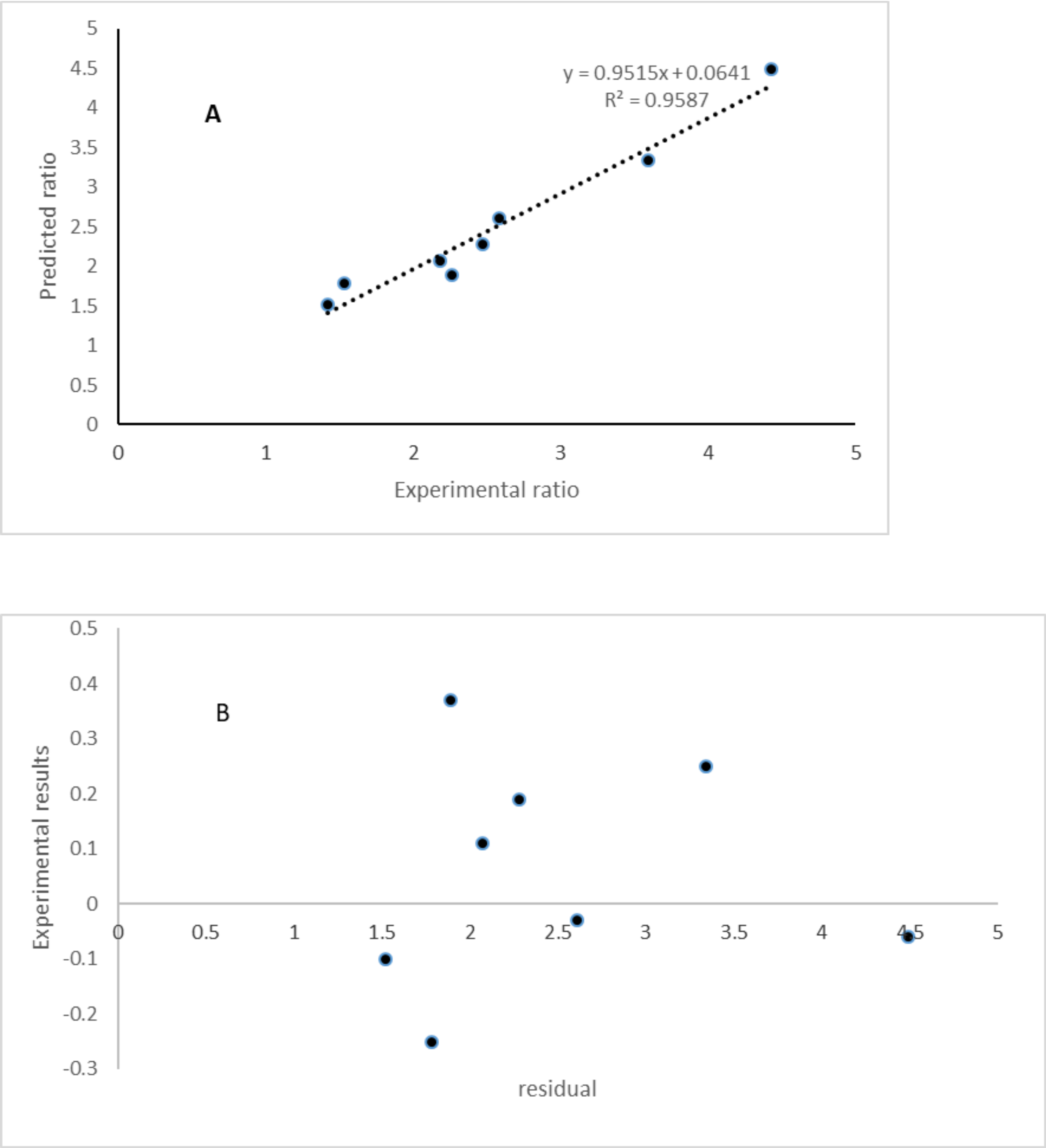

Figure S1. The correlation between the model predictions and the A) observed response and B) the residual plot for freeze-drying process.

Freeze-drying of PEG-PBCL$_9$ NPs using PEG as cryoprotectant

Free PEG was used as the cryoprotectant after PEG-PBCL$_9$ NP preparation. We first assessed the effect of PEG addition on the average diameter and PDI of NPs before freeze-drying. As shown in Figure S2A, after the addition of PEG to the PEG-PBCL$_9$ NPs, the average size of NPs increased from 36.9 $\pm$0.14 nm to ~40.0 nm ($p<0.05$, two-way ANOVA). However, the PDI did not significantly change ($p>0.05$, Two-way ANOVA) except for the addition of 4:1 w/w ratio of PEG 3350 and 8000 Da to PEG-PBCL$_9$ which led to a significant increase in the PDI ($p<0.05$, two-way ANOVA) (Figure S2B) (Table S2.)

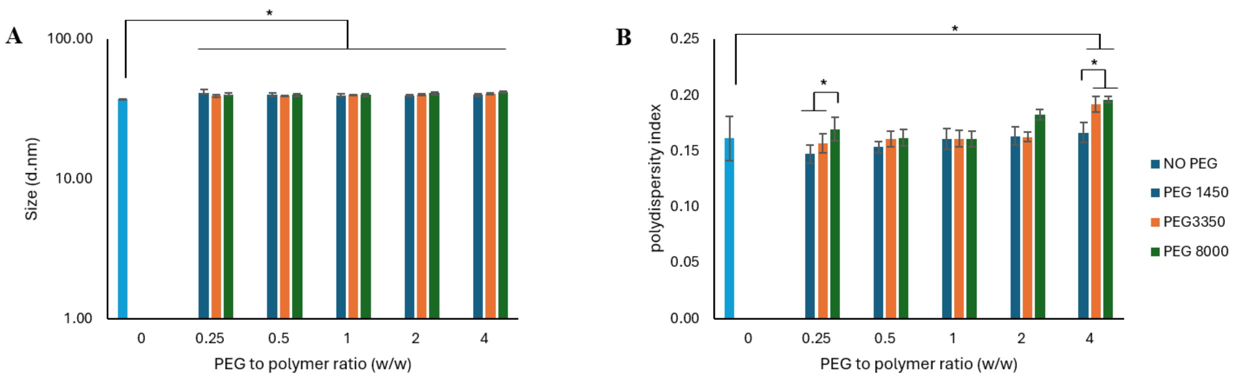

Figure S2. A) PEG-PBCL$_9$ NPs' size and **B)** PDI after the addition of different molecular weight PEGs at different concentrations before freeze-drying. All samples were measured at RT. (* represents $p<0.05$, two-way ANOVA, n=3)

In the next step, the effect of PEG molecular weight, end group and its ratio (w/w) to that of PEG-PBCL$_9$ on the average diameter and polydispersity index of freeze-dried and reconstituted NPs was assessed and compared to that for NPs without PEG addition before freeze-drying (Figure S3). The results showed NPs' average diameter and PDI increase after freeze-drying for all samples including those that contained PEGs of different MWs and end groups at various ratios. Among different PEG MWs, PEG 3350 and 8000 Da seemed to be more efficient cryo-protectants compared to PEG 1450 Da. The least degree of aggregation in reconstituted NPs was observed when PEG 3350 and 8000 Da were added to PEG-PBCL$_9$ NPs, particularly at w/w ratios of 2 and 4 (Figure S3A). The increase in average diameter of NPs was lower for the PEG3350 and 8000 Da (sf/si of ~1.5 at 4:1 PEG to polymer ratio) compared to that for PEG 1450 Da (Sf/Si of 1.8) (Figure S3A). Similar to the average diameter, the PDI of NPs was the lowest for PEG 3350 and 8000 Da

as cryo-protectant at a w/w ratio of 4:1 (Figure S3B). The PDI of PEG-$PBCL_9$ NPs showed a decreasing trend as the weight ratio of PEG as cryo-protectant was increased. This decreasing trend in PDI was observed for all PEG cryo-protectants under study (Figure S3B) (Table S2 and S3).

To study the effect of PEG end group on its cryoprotectant properties, methoxy PEGs with two molecular weights of 2000 and 5000 Da were used as cryo-protectants during freeze-drying of PEG-$PBCL_9$ NPs. Similar to what was observed for the OH-terminated PEGs, the addition of methoxy-terminated PEGs was not able to completely prevent the aggregation of PEG-PBCL NPs upon reconstitution (Figure S3C and D). In other words, irrespective of the MW and ratio of methoxy PEG added as a cryoprotectant, PEG-$PBCL_9$ NPs showed an increase in average diameter and PDI following freeze-drying and reconstitution. However, the degree of this increase was less compared to the PEG-$PBCL_9$ NPs for which no cryoprotectant was used (Sf/Si ratio of 1.4 for 4: 1 w/w ratio of methoxy PEG 5000 to PEG-PBCL versus 3.3 without any cryoprotectant).

Among different ratios, the NPs' size was the lowest for PEG: PEG-$PBCL_9$ w/w ratio of ≥ 2, irrespective of PEG MW (Table S2-S4). The PDI of NPs seemed to decrease significantly as the w/w ratio of PEG to PBCL was increased ≥ 0.25 irrespective of PEG MW although the dose-dependent decrease in PDI seemed steeper for PEG 5000 compared to 2000 Da (Table S4.).

Overall, the best results in terms of inhibition of NP aggregation appeared to be achieved by PEG 3350 and 8000 Da as well as methoxy PEG 5000 Da at 4:1 w/w ratio (Table S3 and S4).

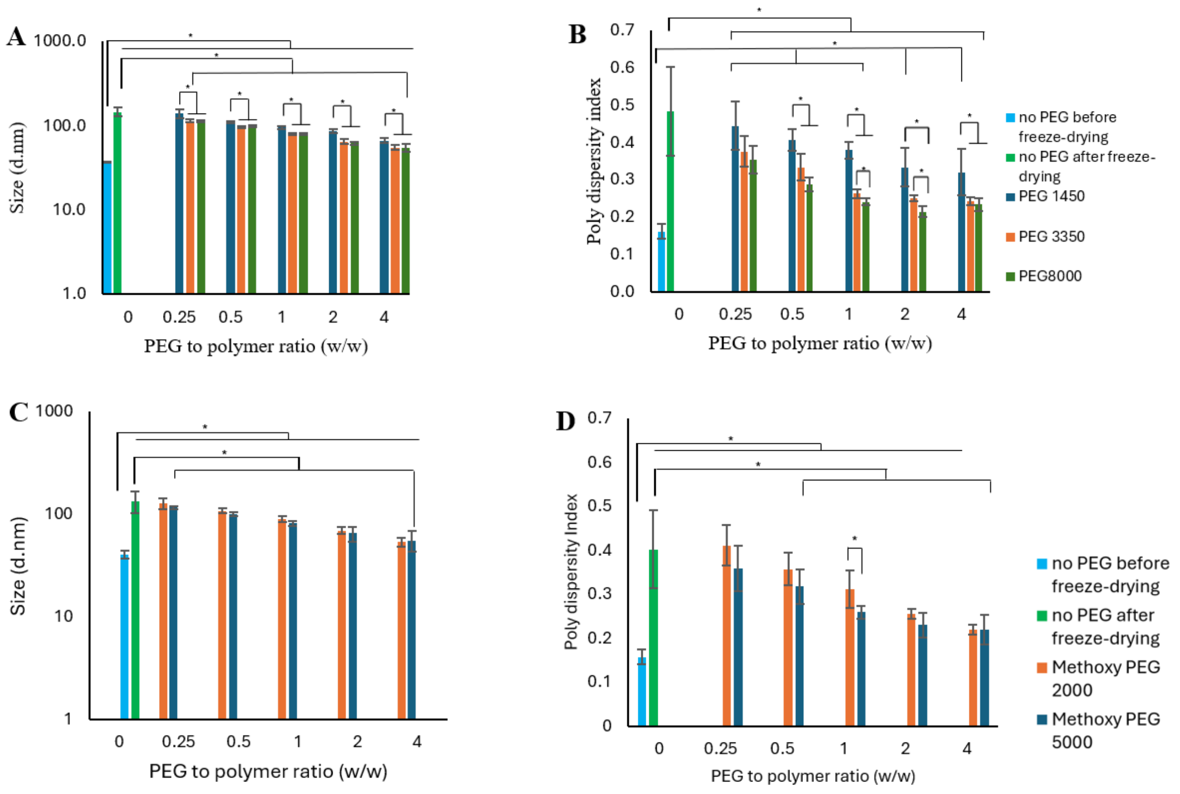

Figure S3. A & C) PEG-PBCL$_9$ NPs' average diameter and **B & D)** PDI after freeze-drying and reconstitution in DiH_2O using **A & B)** hydroxyl terminated PEGs and **C & D)** methoxy terminated PEG at different concentrations and MWs as cryoprotectantt (* represents $p<0.05$, two-way ANOVA, n=3)

Freeze-thawing of PEG-PBCL$_9$ NPs using PEG or sucrose as cryoprotectant

The effect of freezing conditions on the average diameter of PEG-PBCL$_9$ NPs thawed to RT without any cryoprotectant is shown in Figure S4. The average diameter of NPs at RT was 37.3±0.46 nm and the PDI was 0.16±0.01. After freeze-thaw the NPs' average size significantly increased to 57.1±3.88, 60.2±1.14, and 68.6±5.65 nm for NPs that were frozen using liquid nitrogen, -80 °C, and -20 °C freezer, respectively ($p<0.05$, One-way ANOVA, compared to NP size before freeze-thaw). Accordingly, the PDI of NPs significantly increased from 0.16±0.01 at RT to 0.20±0.01, 0.19±0.01, and 0.28±0.02 for the same conditions, respectively ($p<0.05$, One-way ANOVA, compared to PDI of NPs before freeze-thaw). Moreover, the NPs' average diameter and PDI was significantly higher for NPs that were frozen at -20 °C compared to the ones frozen either in liquid nitrogen or the -80 °C freezer ($p<0.05$, One-way ANOVA). However, these values

did not significantly change between NPs that were frozen in liquid nitrogen and the -80 °C freezer ($p>0.05$, One-way ANOVA) (Table S9.)

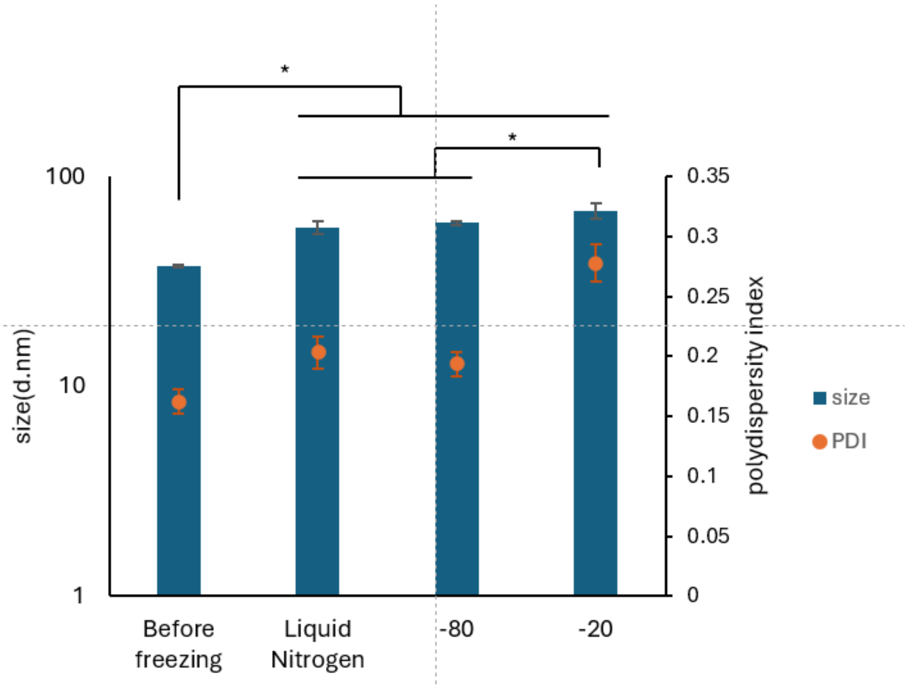

Figure S4. PEG-PBCL9 NPs' average diameter and PDI after freeze-thaw at different freezing conditions (* represents $p<0.05$, One-way ANOVA, n=3)

In the next step, PEGs of different MW and end groups as well as sucrose were used as cryoprotectant. The w/w ratio of PEG was 2:1 while sucrose concentration was 13.25:1 to PEG-PBCL9. As illustrated in Figure S5 and Table S10, for PEG-PBCL9, the NPs' average diameter significantly increased by 1.80 folds when no cryoprotectant was added ($p<0.05$, One-way ANOVA). NPs' average diameter when sucrose was used as a cryoprotectant was significantly higher than those before freeze-thawing (1.20 fold increase), but this value was lower than the NPs without any cryoprotectant ($p<0.05$, One-way ANOVA). Addition of PEG seem to reduce the PDI of NPs compared to the ones with no cryoprotectant ($p<0.05$, One-way ANOVA). Sucrose seems to lower the PDI of PEG-PBCL9 NPs compared to when no cryoprotectant was added during freeze-thaw, but was not as efficient as PEGs in this regard (Figure S5, Tables S10 & S11)

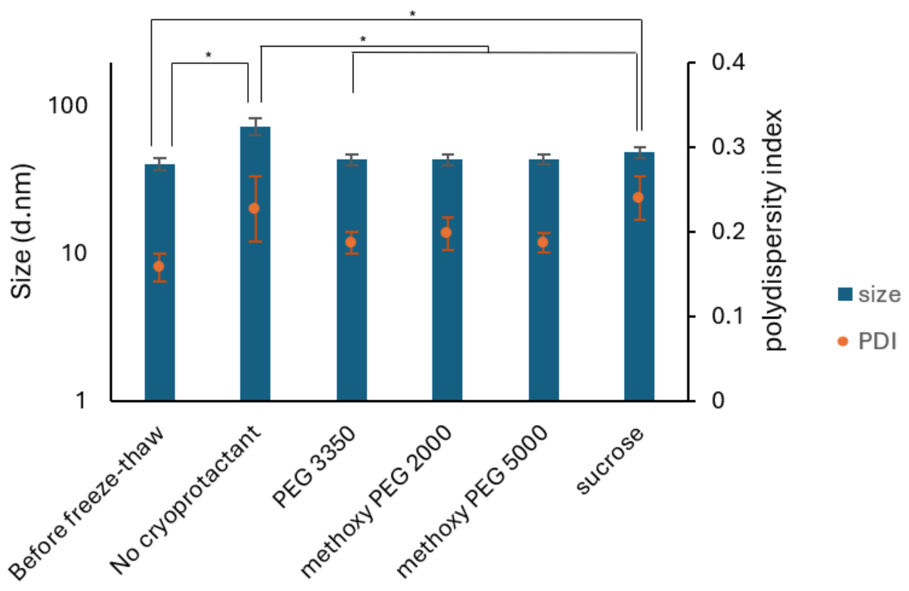

Figure S5. PEG-PBCL$_9$ NPs' average diameter and PDI before and after freeze-thawing without or with PEGs or sucrose as cryoprotectant. The ratio of PEGs and sucrose to PEG-PBCL$_9$ were 2:1 and 13.25:1 w/w, respectively. (* represent $p<0.05$, One-way ANOVA, n=3).

Table S2. Size distribution of PEO-PBCL$_9$ NPs after addition of different Mw of PEG at room temperature

	1:0.25	1:0.5	1:1	1:2	1:4
PEG 1450	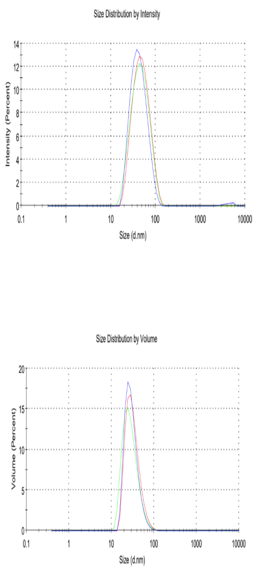	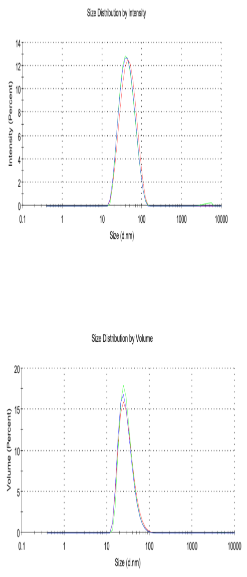	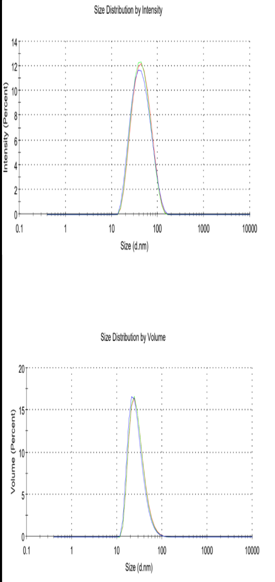	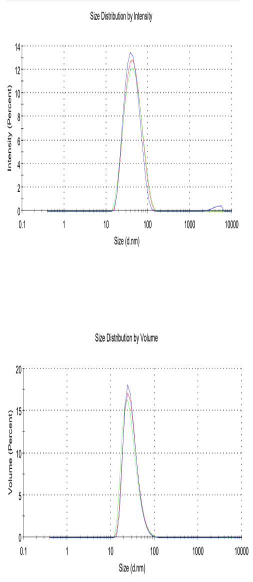	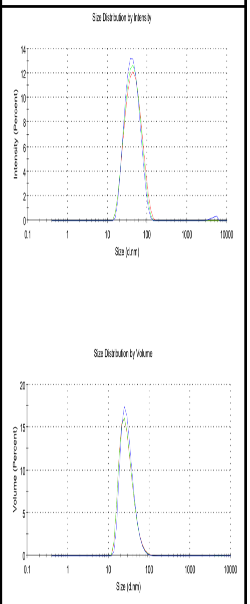
PEG 3350	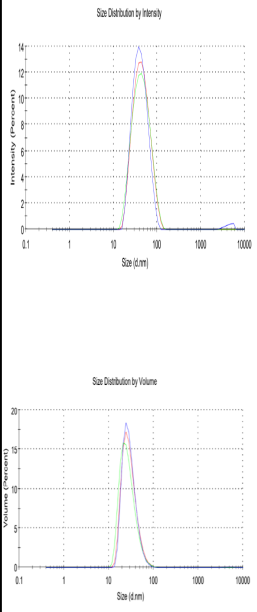	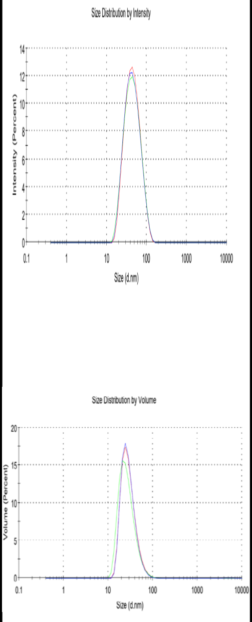	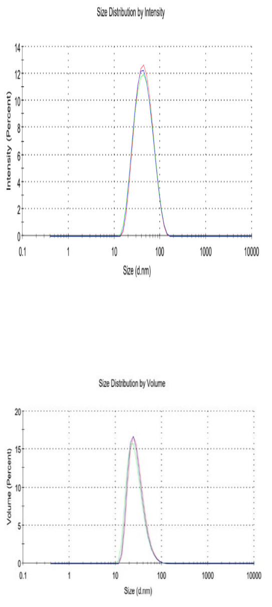	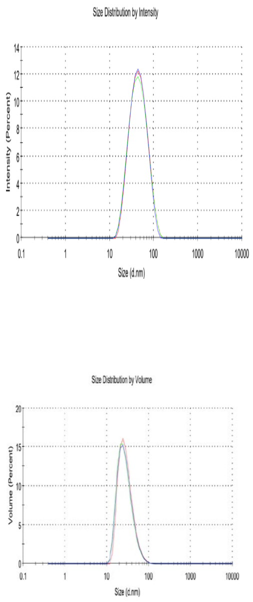	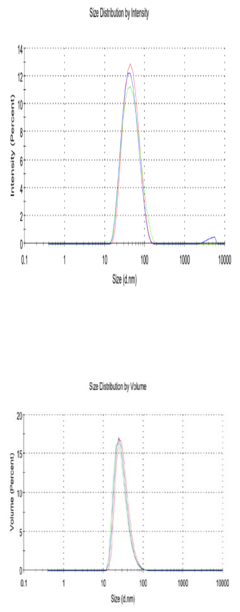
PEG 8000	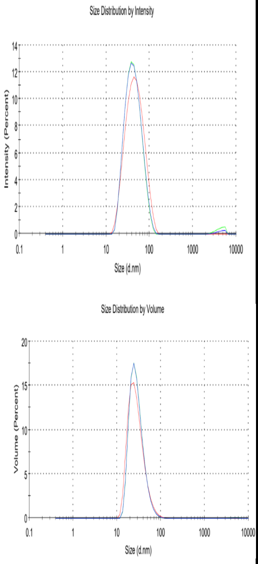	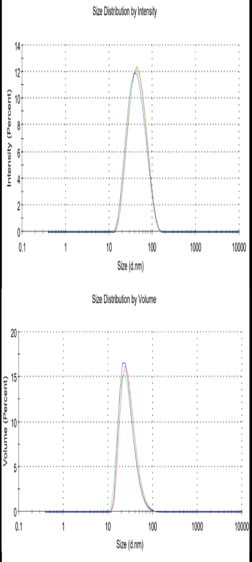	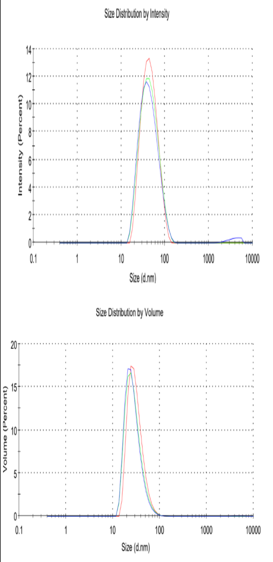	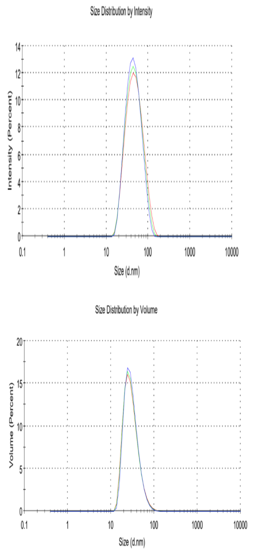	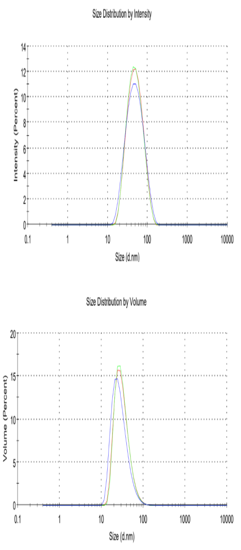

Table S3. Size distribution of PEO-PBCL$_9$ NPs after freeze-drying with different ratios and Mw of PEG

	0.25:1	0.5:1	1:1	2:1	4:1
PEG 1450	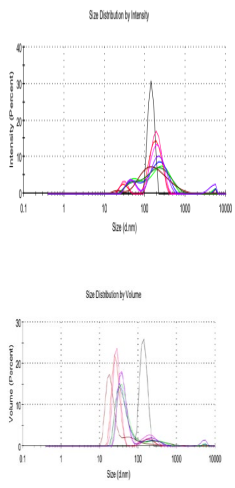	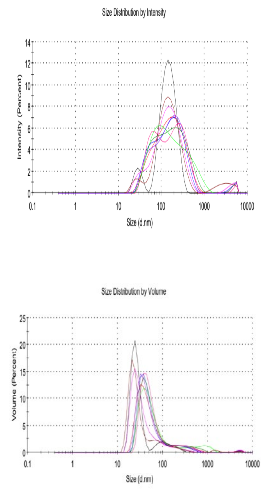	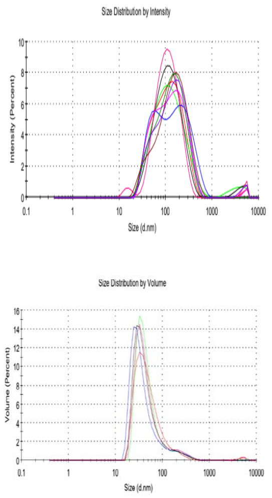	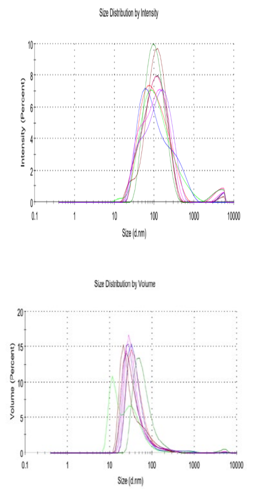	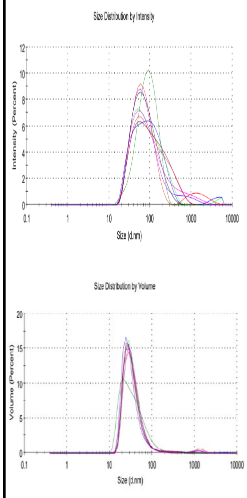
PEG 3350	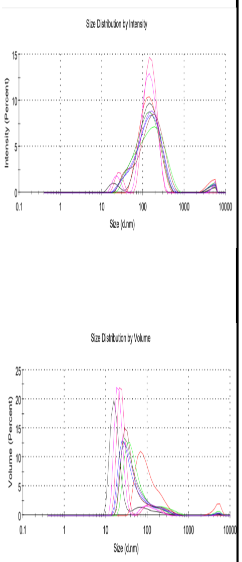	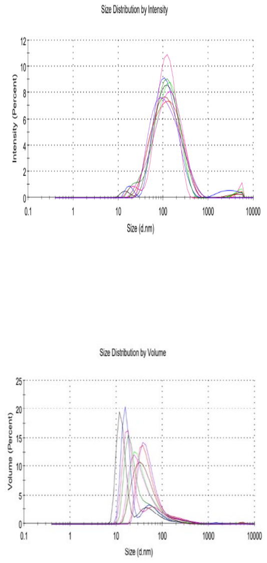	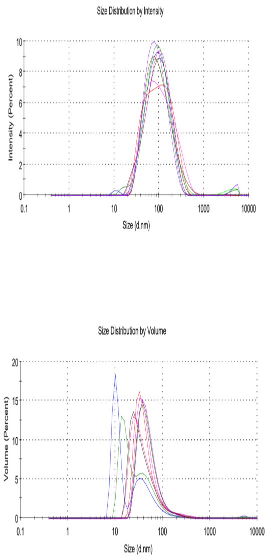	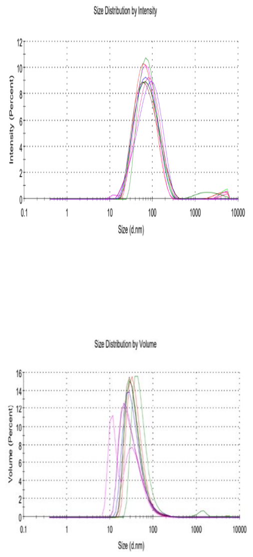	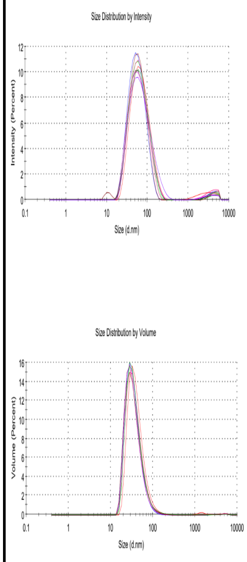
PEG 8000	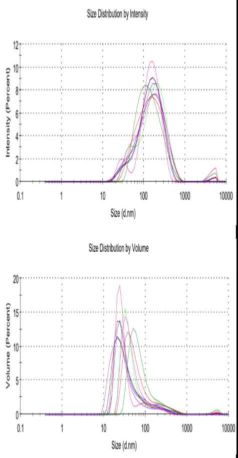	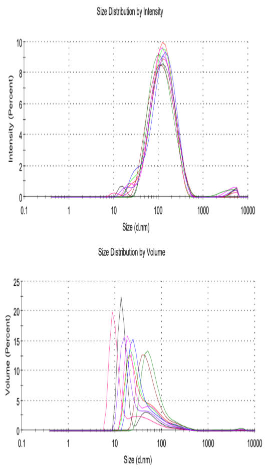	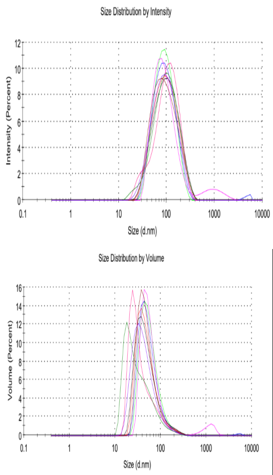	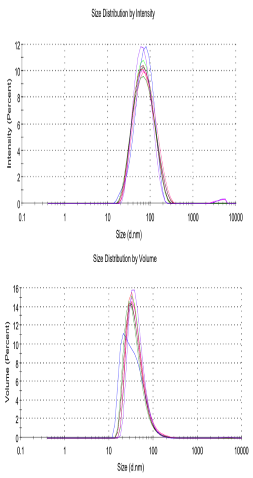	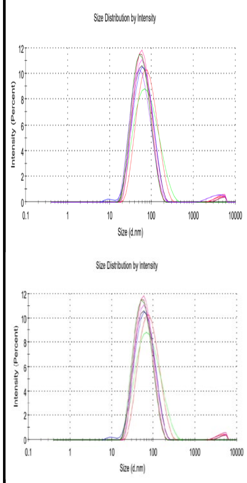
No cryoprotectant	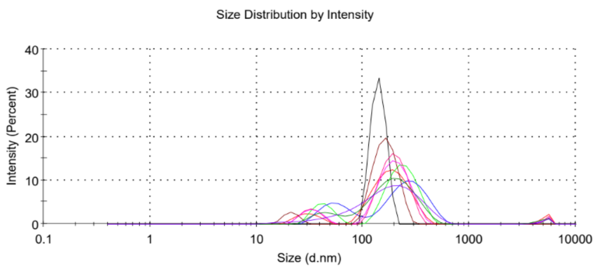		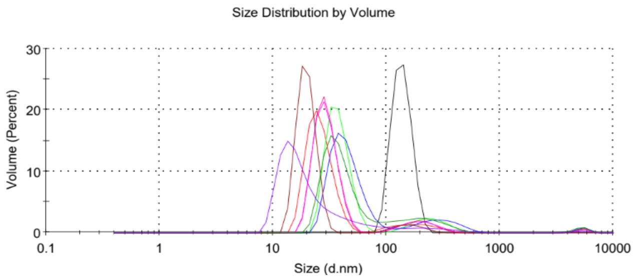		

Table S4. Size distribution of PEO-PBCL$_9$ NPs after freeze-drying with different ratios and Mw of methoxy-PEG

Methoxy-PEG 2000

0.25:1

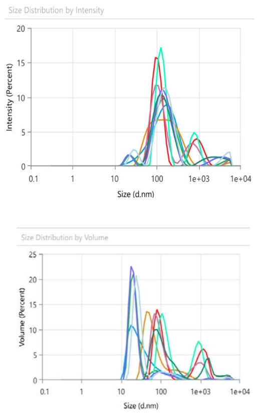

Name	Mean	Standard Deviation
Z-Average (nm)	127.5	15
Polydispersity Index (PI)	0.4119	0.04627
Intercept	0.9647	0.004536
Fit Error	0.003325	0.001059
In Range (%)	94.07	2.416
Peak One Mean by Intensity (nm)	159	45.74
Peak Two Mean by Intensity (nm)	1498	1729
Peak Three Mean by Intensity (nm)	3674	2056

0.5:1

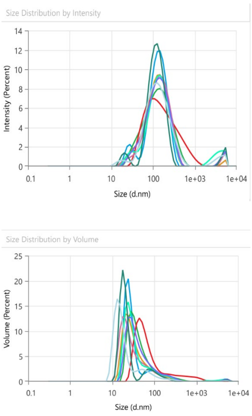

Name	Mean	Standard Deviation
Z-Average (nm)	108.4	6.268
Polydispersity Index (PI)	0.3576	0.03691
Intercept	0.9629	0.004686
Fit Error	0.003114	0.0004736
In Range (%)	94.65	1.132
Peak One Mean by Intensity (nm)	153.9	24.57
Peak Two Mean by Intensity (nm)	3202	2216
Peak Three Mean by Intensity (nm)	1642	2802

1:1

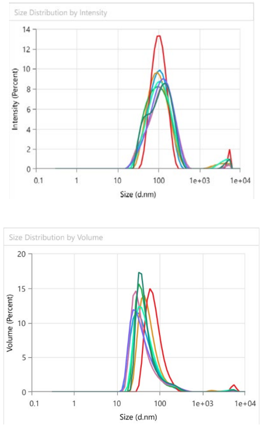

Name	Mean	Standard Deviation
Z-Average (nm)	90.04	6.226
Polydispersity Index (PI)	0.312	0.0437
Intercept	0.9626	0.006386
Fit Error	0.002145	0.0002224
In Range (%)	95.54	1.734
Peak One Mean by Intensity (nm)	121.6	11.5
Peak Two Mean by Intensity (nm)	4532	637.5

2:1

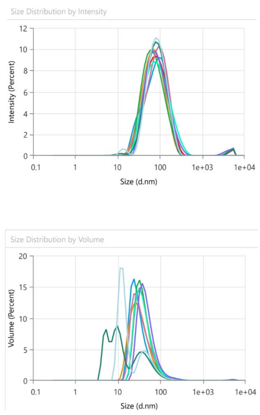

Name	Mean	Standard Deviation
Z-Average (nm)	69.72	4.852
Polydispersity Index (PI)	0.2565	0.0109
Intercept	0.97	0.003015
Fit Error	0.001893	0.0002418
In Range (%)	97.05	0.4748
Peak One Mean by Intensity (nm)	92.63	7.364
Peak Two Mean by Intensity (nm)	3697	2070
Peak Three Mean by Intensity (nm)	12.44	-

4:1

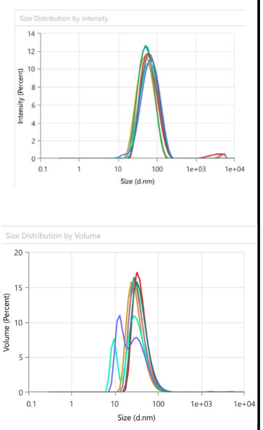

Name	Mean	Standard Deviation
Z-Average (nm)	53.91	5.374
Polydispersity Index (PI)	0.2198	0.01182
Intercept	0.9711	0.008586
Fit Error	0.001382	0.0002262
In Range (%)	97.44	0.494
Peak One Mean by Intensity (nm)	66.34	7.875
Peak Two Mean by Intensity (nm)	3566	2073

Methoxy-PEG 5000

0.25:1

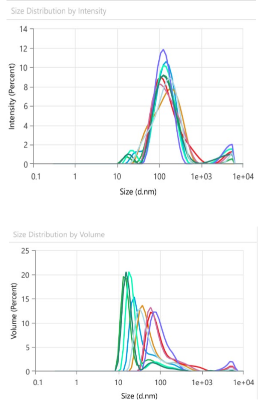

Name	Mean	Standard Deviation
Z-Average (nm)	116.3	4.657
Polydispersity Index (PI)	0.3584	0.05191
Intercept	0.9695	0.008229
Fit Error	0.003449	0.000422
In Range (%)	95.43	0.639
Peak One Mean by Intensity (nm)	164.3	16.93
Peak Two Mean by Intensity (nm)	3619	1653
Peak Three Mean by Intensity (nm)	1553	2654

0.5:1

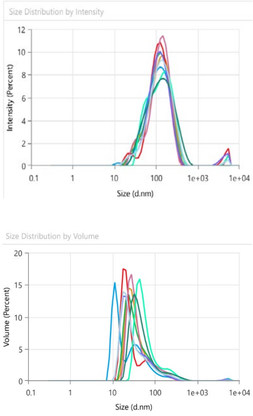

Name	Mean	Standard Deviation
Z-Average (nm)	100.6	4.746
Polydispersity Index (PI)	0.3179	0.03906
Intercept	0.9685	0.009066
Fit Error	0.002899	0.0004922
In Range (%)	96.29	0.4698
Peak One Mean by Intensity (nm)	140	9.062
Peak Two Mean by Intensity (nm)	2383	2593
Peak Three Mean by Intensity (nm)	4975	333.4

1:1

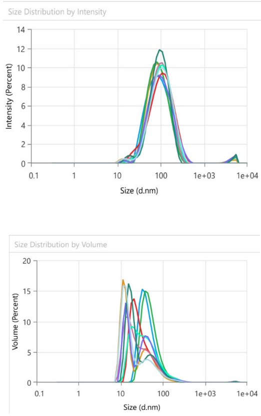

Name	Mean	Standard Deviation
Z-Average (nm)	81.68	4.68
Polydispersity Index (PI)	0.2593	0.0138
Intercept	0.9706	0.005241
Fit Error	0.00216	0.0003302
In Range (%)	96.87	0.5474
Peak One Mean by Intensity (nm)	108.1	9.43
Peak Two Mean by Intensity (nm)	2358	2565
Peak Three Mean by Intensity (nm)	3100	2693

2:1

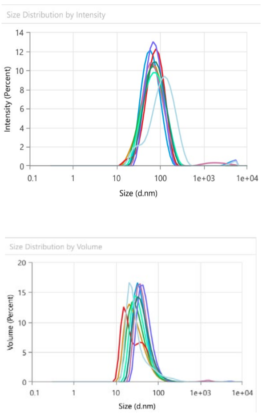

Name	Mean	Standard Deviation
Z-Average (nm)	65.29	10.76
Polydispersity Index (PI)	0.2301	0.02764
Intercept	0.9773	0.005712
Fit Error	0.001697	0.0008386
In Range (%)	97.14	0.3502
Peak One Mean by Intensity (nm)	83.84	17.9
Peak Two Mean by Intensity (nm)	3998	1211

4:1

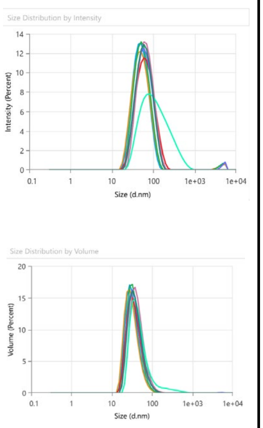

Name	Mean	Standard Deviation
Z-Average (nm)	55.73	12.58
Polydispersity Index (PI)	0.2189	0.03376
Intercept	0.9754	0.009968
Fit Error	0.001427	0.00058
In Range (%)	97.43	0.4397
Peak One Mean by Intensity (nm)	71.53	28.26
Peak Two Mean by Intensity (nm)	4713	210.7

No PEG added

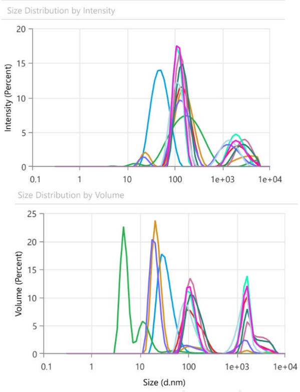

Name	Mean	Standard Deviation
Z-Average (nm)	135.1	32.31
Polydispersity Index (PI)	0.4028	0.08885
Intercept	0.9657	0.008888
Fit Error	0.003815	0.001315
In Range (%)	95.2	1.009
Peak One Mean by Intensity (nm)	137.1	40.89
Peak Two Mean by Intensity (nm)	2593	884.3
Peak Three Mean by Intensity (nm)	20.74	4.783

Table S5. Size distribution of PEO-PBCL$_{22}$ NPs after freeze-drying with and with w/w 2:1 of PEG: polymer and 13.25:1 of sucrose to polymer Mw of PEG

Cryoprotectant	Size distribution

No cryoprotectant before freeze-drying

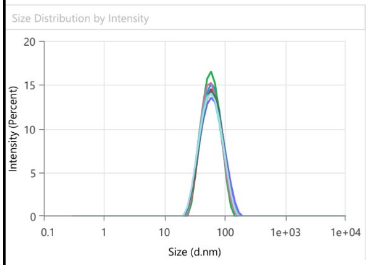

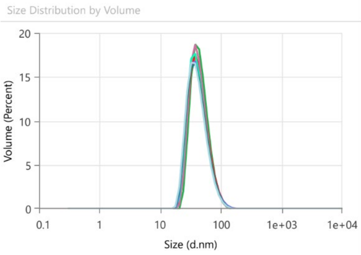

Name	Mean	Standard Deviation
Z-Average (nm)	55.43	0.9756
Polydispersity Index (PI)	0.1223	0.01652
Intercept	0.9606	0.01376
Fit Error	0.0005088	0.0001278
In Range (%)	97.57	0.2746
Peak One Mean by Intensity (nm)	63.65	1.534

No cryoprotectant After freeze - drying

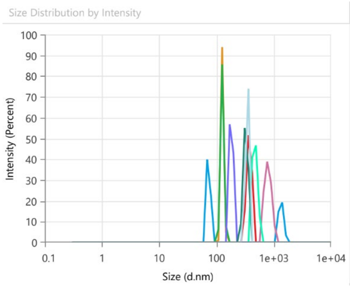

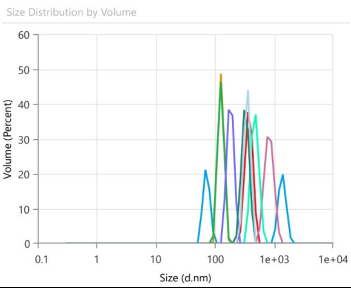

Name	Mean	Standard Deviation
Z-Average (nm)	1280	549.5
Polydispersity Index (PI)	0.8748	0.1696
Intercept	0.9092	0.02302
Fit Error	0.01892	0.01416
In Range (%)	87.45	4.524
Peak One Mean by Intensity (nm)	313.8	226.8
Peak Two Mean by Intensity (nm)	1348	-

PEG 3350

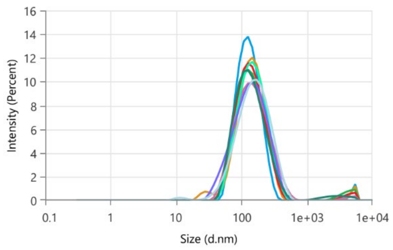

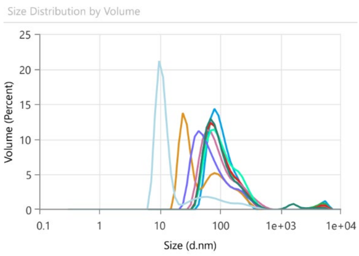

Name	Mean	Standard Deviation
Z-Average (nm)	124.5	4.933
Polydispersity Index (PI)	0.2554	0.01179
Intercept	0.9659	0.00815
Fit Error	0.001996	0.0004257
In Range (%)	95.08	1.038
Peak One Mean by Intensity (nm)	155.5	10.88
Peak Two Mean by Intensity (nm)	3032	2154
Peak Three Mean by Intensity (nm)	5079	-

Methoxy-PEG 2000

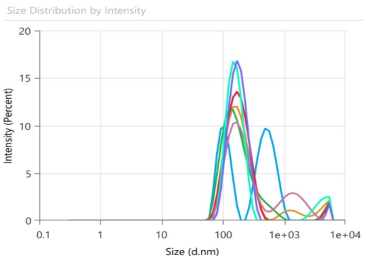

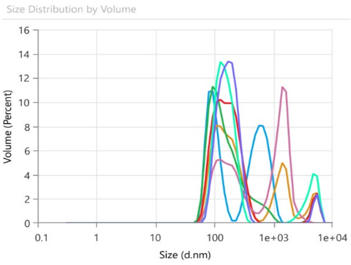

Name	Mean	Standard Deviation
Z-Average (nm)	173.8	12.72
Polydispersity Index (PI)	0.3557	0.04551
Intercept	0.9604	0.01106
Fit Error	0.002813	0.0005993
In Range (%)	94.13	1.144
Peak One Mean by Intensity (nm)	236.1	136.6
Peak Two Mean by Intensity (nm)	3636	2011
Peak Three Mean by Intensity (nm)	1421	-

Methoxy-PEG 5000

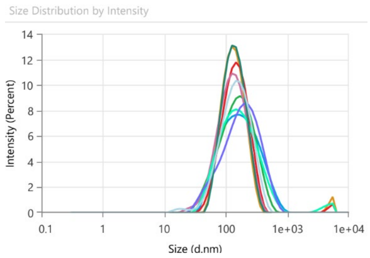

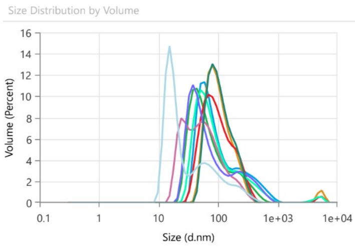

Name	Mean	Standard Deviation
Z-Average (nm)	126	9.683
Polydispersity Index (PI)	0.2657	0.03666
Intercept	0.9694	0.005502
Fit Error	0.002327	0.0006552
In Range (%)	96	0.7891
Peak One Mean by Intensity (nm)	171.3	27.89
Peak Two Mean by Intensity (nm)	3629	2423

Sucrose

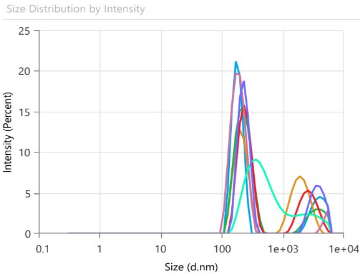

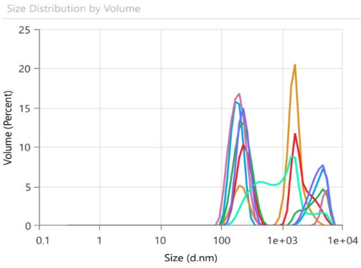

Name	Mean	Standard Deviation
Z-Average (nm)	287.9	68.66
Polydispersity Index (PI)	0.4229	0.06861
Intercept	0.9513	0.0103
Fit Error	0.002632	0.0008875
In Range (%)	94.67	0.6204
Peak One Mean by Intensity (nm)	254.7	107.4
Peak Two Mean by Intensity (nm)	3415	976.1

Table S6. Size distribution of PEO-PBCL$_{22}$ NPs after freeze-drying with and with w/w 4:1 of PEG: polymer and 13.25:1 of sucrose to polymer Mw of PEG

<table>
<tr><th>Cryoprotectant</th><th>Size distribution</th></tr>
<tr><td>No cryoprotectant before freeze-drying</td><td>
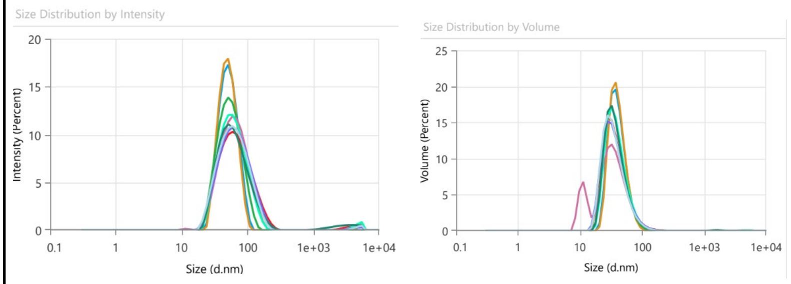

<table>
<tr><th>Name</th><th>Mean</th><th>Standard Deviation</th></tr>
<tr><td>Z-Average (nm)</td><td>54.5</td><td>3.67</td></tr>
<tr><td>Polydispersity Index (PI)</td><td>0.2013</td><td>0.03523</td></tr>
<tr><td>Intercept</td><td>0.9655</td><td>0.01058</td></tr>
<tr><td>Fit Error</td><td>0.0007165</td><td>0.0001743</td></tr>
<tr><td>In Range (%)</td><td>94.75</td><td>1.434</td></tr>
<tr><td>Peak One Mean by Intensity (nm)</td><td>63.83</td><td>7.984</td></tr>
<tr><td>Peak Two Mean by Intensity (nm)</td><td>4409</td><td>661.7</td></tr>
<tr><td>Peak Three Mean by Intensity (nm)</td><td>11.82</td><td>-</td></tr>
</table>
</td></tr>
<tr><td>No cryoprotectant After freeze - drying</td><td>
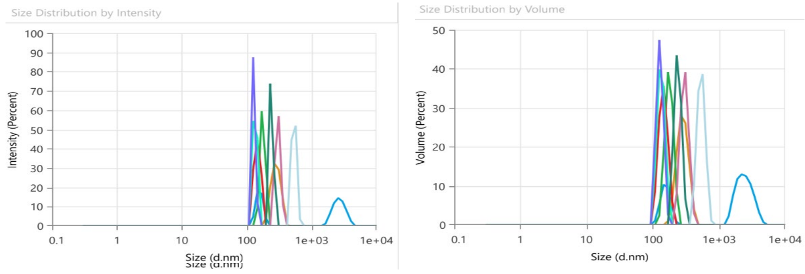

<table>
<tr><th>Name</th><th>Mean</th><th>Standard Deviation</th></tr>
<tr><td>Z-Average (nm)</td><td>1455</td><td>1016</td></tr>
<tr><td>Polydispersity Index (PI)</td><td>0.8627</td><td>0.2339</td></tr>
<tr><td>Intercept</td><td>0.9213</td><td>0.02003</td></tr>
<tr><td>Fit Error</td><td>0.01451</td><td>0.007413</td></tr>
<tr><td>In Range (%)</td><td>87.59</td><td>3.034</td></tr>
<tr><td>Peak One Mean by Intensity (nm)</td><td>514.7</td><td>824.1</td></tr>
<tr><td>Peak Two Mean by Intensity (nm)</td><td>157.6</td><td>-</td></tr>
</table>
</td></tr>
<tr><td>PEG 3350</td><td>
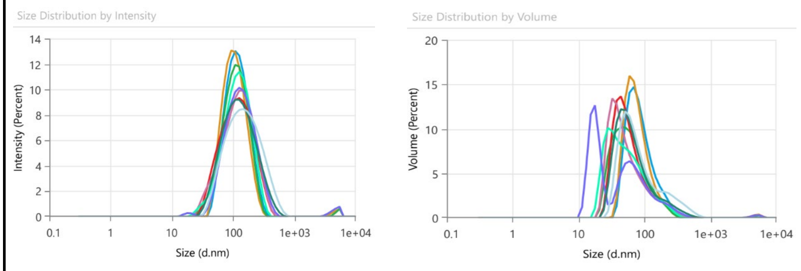

<table>
<tr><th>Name</th><th>Mean</th><th>Standard Deviation</th></tr>
<tr><td>Z-Average (nm)</td><td>105.8</td><td>8.054</td></tr>
<tr><td>Polydispersity Index (PI)</td><td>0.2359</td><td>0.03758</td></tr>
<tr><td>Intercept</td><td>0.9692</td><td>0.003214</td></tr>
<tr><td>Fit Error</td><td>0.001574</td><td>0.0006149</td></tr>
<tr><td>In Range (%)</td><td>96.45</td><td>0.5029</td></tr>
<tr><td>Peak One Mean by Intensity (nm)</td><td>136.3</td><td>19.19</td></tr>
<tr><td>Peak Two Mean by Intensity (nm)</td><td>4825</td><td>244.7</td></tr>
<tr><td>Peak Three Mean by Intensity (nm)</td><td>19.39</td><td>-</td></tr>
</table>
</td></tr>
<tr><td>Methoxy-PEG 2000</td><td>
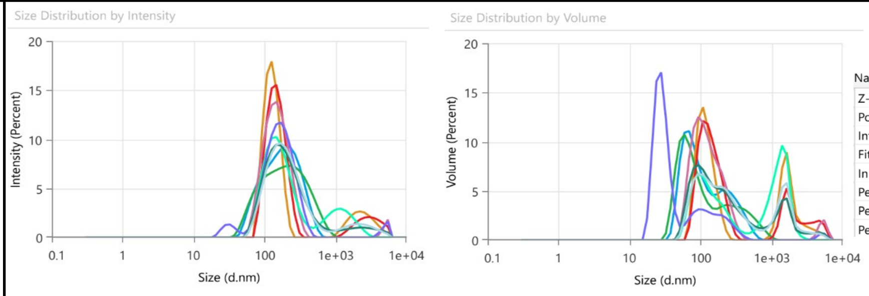

<table>
<tr><th>Name</th><th>Mean</th><th>Standard Deviation</th></tr>
<tr><td>Z-Average (nm)</td><td>155.3</td><td>10.93</td></tr>
<tr><td>Polydispersity Index (PI)</td><td>0.3338</td><td>0.03878</td></tr>
<tr><td>Intercept</td><td>0.9623</td><td>0.004808</td></tr>
<tr><td>Fit Error</td><td>0.002045</td><td>0.0005968</td></tr>
<tr><td>In Range (%)</td><td>95.41</td><td>1.595</td></tr>
<tr><td>Peak One Mean by Intensity (nm)</td><td>182.9</td><td>35.81</td></tr>
<tr><td>Peak Two Mean by Intensity (nm)</td><td>2412</td><td>1567</td></tr>
<tr><td>Peak Three Mean by Intensity (nm)</td><td>4946</td><td>-</td></tr>
</table>
</td></tr>
<tr><td>Methoxy-PEG 5000</td><td>
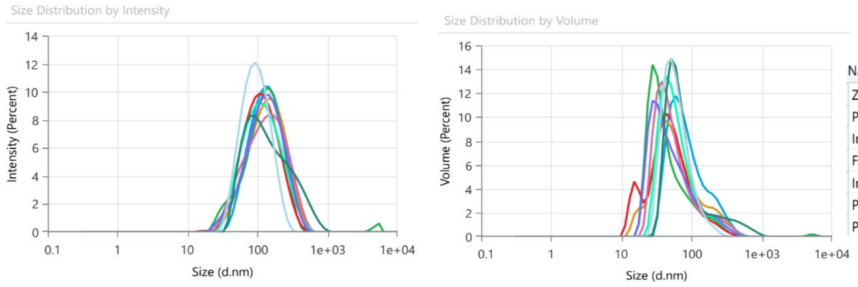

<table>
<tr><th>Name</th><th>Mean</th><th>Standard Deviation</th></tr>
<tr><td>Z-Average (nm)</td><td>105.3</td><td>11.05</td></tr>
<tr><td>Polydispersity Index (PI)</td><td>0.2503</td><td>0.03259</td></tr>
<tr><td>Intercept</td><td>0.9655</td><td>0.004272</td></tr>
<tr><td>Fit Error</td><td>0.001942</td><td>0.0006092</td></tr>
<tr><td>In Range (%)</td><td>96.28</td><td>0.7596</td></tr>
<tr><td>Peak One Mean by Intensity (nm)</td><td>144.4</td><td>21.97</td></tr>
<tr><td>Peak Two Mean by Intensity (nm)</td><td>2489</td><td>3496</td></tr>
</table>
</td></tr>
<tr><td>Sucrose</td><td>
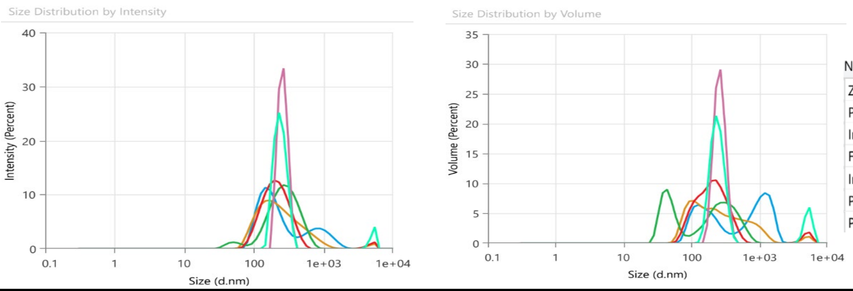

<table>
<tr><th>Name</th><th>Mean</th><th>Standard Deviation</th></tr>
<tr><td>Z-Average (nm)</td><td>229.9</td><td>51.13</td></tr>
<tr><td>Polydispersity Index (PI)</td><td>0.319</td><td>0.06412</td></tr>
<tr><td>Intercept</td><td>0.9578</td><td>0.005978</td></tr>
<tr><td>Fit Error</td><td>0.003677</td><td>0.001921</td></tr>
<tr><td>In Range (%)</td><td>95.49</td><td>0.3308</td></tr>
<tr><td>Peak One Mean by Intensity (nm)</td><td>247.6</td><td>41.81</td></tr>
<tr><td>Peak Two Mean by Intensity (nm)</td><td>3147</td><td>2465</td></tr>
</table>
</td></tr>
</table>

Table S7. Size distribution of PEO-PBCL$_{22}$ NPs loaded with A83B4C63 after freeze-drying with 4:1 ratio of methoxy-PEG 5000 to polymer

<table>
<tr><th>Cryoprotectant</th><th>Size distribution</th></tr>
<tr><td>No cryoprotectant before freeze-drying</td><td>
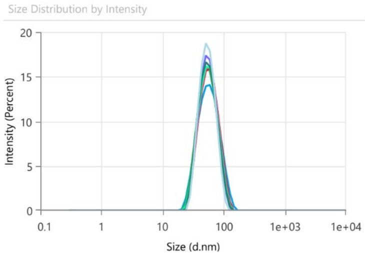

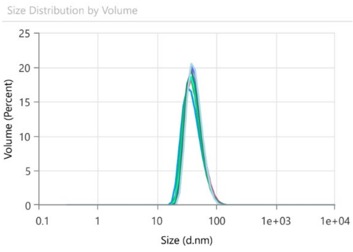

<table>
<tr><th>Name</th><th>Mean</th><th>Standard Deviation</th></tr>
<tr><td>Z-Average (nm)</td><td>51.87</td><td>0.8711</td></tr>
<tr><td>Polydispersity Index (PI)</td><td>0.09432</td><td>0.01254</td></tr>
<tr><td>Intercept</td><td>0.9659</td><td>0.003732</td></tr>
<tr><td>Fit Error</td><td>0.0008437</td><td>0.0001947</td></tr>
<tr><td>In Range (%)</td><td>97.3</td><td>0.2482</td></tr>
<tr><td>Peak One Mean by Intensity (nm)</td><td>57.85</td><td>1.507</td></tr>
</table>
</td></tr>
<tr><td>No cryoprotectant After freeze - drying</td><td>
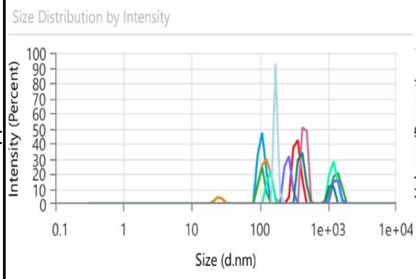

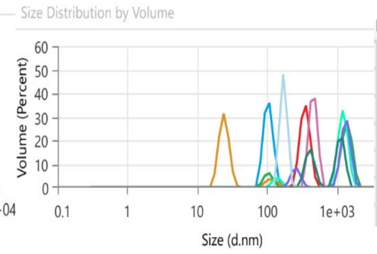

<table>
<tr><th>Name</th><th>Mean</th><th>Standard Deviation</th></tr>
<tr><td>Z-Average (nm)</td><td>1212</td><td>526.3</td></tr>
<tr><td>Polydispersity Index (PI)</td><td>0.8705</td><td>0.1454</td></tr>
<tr><td>Intercept</td><td>0.8623</td><td>0.03104</td></tr>
<tr><td>Fit Error</td><td>0.01936</td><td>0.009538</td></tr>
<tr><td>In Range (%)</td><td>86.72</td><td>3.672</td></tr>
<tr><td>Peak One Mean by Intensity (nm)</td><td>490.2</td><td>459.7</td></tr>
<tr><td>Peak Two Mean by Intensity (nm)</td><td>539.2</td><td>619.9</td></tr>
</table>
</td></tr>
<tr><td>Methoxy-PEG 5000 (1:4 polymer to PEG ratio) Before centrifugation</td><td>
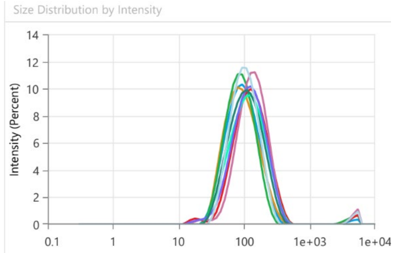

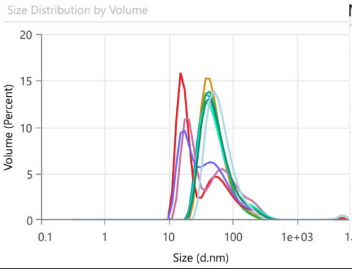

<table>
<tr><th>Name</th><th>Mean</th><th>Standard Deviation</th></tr>
<tr><td>Z-Average (nm)</td><td>95.09</td><td>14.55</td></tr>
<tr><td>Polydispersity Index (PI)</td><td>0.2418</td><td>0.01559</td></tr>
<tr><td>Intercept</td><td>0.9611</td><td>0.01124</td></tr>
<tr><td>Fit Error</td><td>0.001617</td><td>0.000408</td></tr>
<tr><td>In Range (%)</td><td>96.59</td><td>0.5794</td></tr>
<tr><td>Peak One Mean by Intensity (nm)</td><td>121.9</td><td>18.27</td></tr>
<tr><td>Peak Two Mean by Intensity (nm)</td><td>3744</td><td>2090</td></tr>
<tr><td>Peak Three Mean by Intensity (nm)</td><td>2387</td><td>3341</td></tr>
</table>
</td></tr>
<tr><td>Methoxy-PEG 5000 (1:4 polymer to PEG ratio) After centrifugation</td><td>
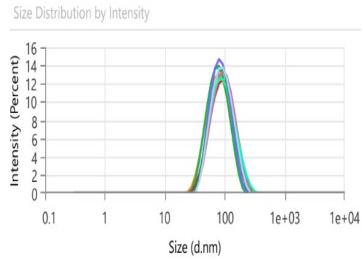

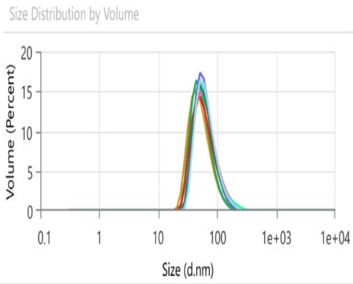

<table>
<tr><th>Name</th><th>Mean</th><th>Standard Deviation</th></tr>
<tr><td>Z-Average (nm)</td><td>78.41</td><td>6.454</td></tr>
<tr><td>Polydispersity Index (PI)</td><td>0.1502</td><td>0.009382</td></tr>
<tr><td>Intercept</td><td>0.9667</td><td>0.004977</td></tr>
<tr><td>Fit Error</td><td>0.0007299</td><td>8.159E-05</td></tr>
<tr><td>In Range (%)</td><td>97.04</td><td>0.2073</td></tr>
<tr><td>Peak One Mean by Intensity (nm)</td><td>92.43</td><td>8.023</td></tr>
</table>
</td></tr>
</table>

Table S8. Size distribution of PEO-PBCL$_{22}$ NPs loaded with A4 after freeze-drying with 4:1 ratio of methoxy-PEG 5000 to polymer

<table>
<tr><th>Cryoprotectant</th><th>Size distribution</th></tr>
<tr><td>No cryoprotectant before freeze-drying</td><td>
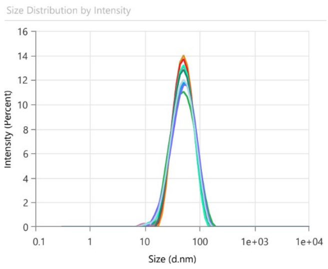

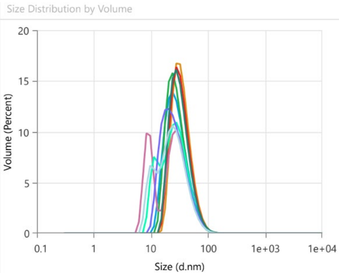

<table>
<tr><th>Name</th><th>Mean</th><th>Standard Deviation</th></tr>
<tr><td>Z-Average (nm)</td><td>44.47</td><td>0.6119</td></tr>
<tr><td>Polydispersity Index (PI)</td><td>0.1707</td><td>0.01369</td></tr>
<tr><td>Intercept</td><td>0.9668</td><td>0.01218</td></tr>
<tr><td>Fit Error</td><td>0.001137</td><td>0.0001183</td></tr>
<tr><td>In Range (%)</td><td>97.64</td><td>0.3382</td></tr>
<tr><td>Peak One Mean by Intensity (nm)</td><td>54.39</td><td>1.378</td></tr>
<tr><td>Peak Two Mean by Intensity (nm)</td><td>9.892</td><td>-</td></tr>
</table>
</td></tr>
<tr><td>No cryoprotectant After freeze - drying</td><td>
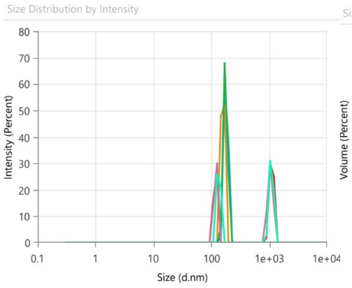

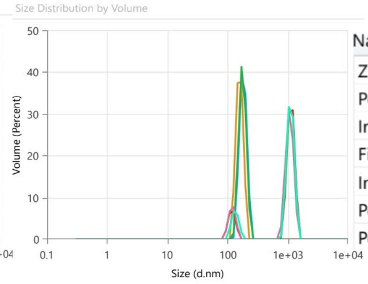

<table>
<tr><th>Name</th><th>Mean</th><th>Standard Deviation</th></tr>
<tr><td>Z-Average (nm)</td><td>1851</td><td>487</td></tr>
<tr><td>Polydispersity Index (PI)</td><td>0.9585</td><td>0.06428</td></tr>
<tr><td>Intercept</td><td>0.8614</td><td>0.02096</td></tr>
<tr><td>Fit Error</td><td>0.01819</td><td>0.004952</td></tr>
<tr><td>In Range (%)</td><td>86.55</td><td>1.906</td></tr>
<tr><td>Peak One Mean by Intensity (nm)</td><td>628.1</td><td>500.7</td></tr>
<tr><td>Peak Two Mean by Intensity (nm)</td><td>124.5</td><td>8.793</td></tr>
</table>
</td></tr>
<tr><td>Methoxy-PEG 5000 (1:4 polymer to PEG ratio) Before centrifugation</td><td>
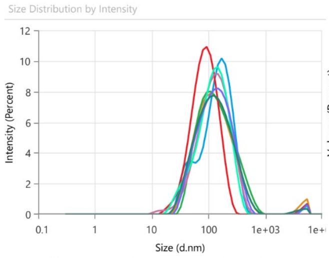

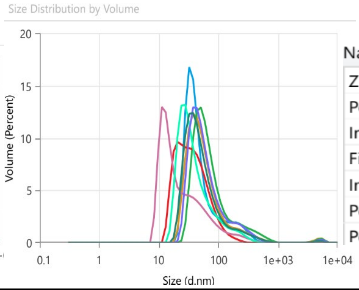

<table>
<tr><th>Name</th><th>Mean</th><th>Standard Deviation</th></tr>
<tr><td>Z-Average (nm)</td><td>102.9</td><td>12.16</td></tr>
<tr><td>Polydispersity Index (PI)</td><td>0.3105</td><td>0.03877</td></tr>
<tr><td>Intercept</td><td>0.9648</td><td>0.003537</td></tr>
<tr><td>Fit Error</td><td>0.002533</td><td>0.0008242</td></tr>
<tr><td>In Range (%)</td><td>96.76</td><td>0.4968</td></tr>
<tr><td>Peak One Mean by Intensity (nm)</td><td>148.5</td><td>24.44</td></tr>
<tr><td>Peak Two Mean by Intensity (nm)</td><td>4092</td><td>1804</td></tr>
</table>
</td></tr>
<tr><td>Methoxy-PEG 5000 (1:4 polymer to PEG ratio) After centrifugation</td><td>
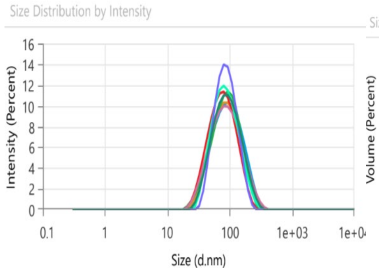

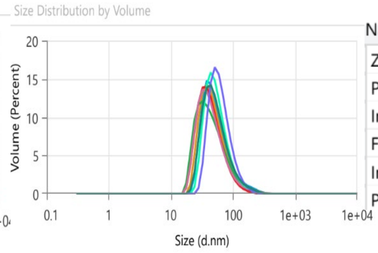

<table>
<tr><th>Name</th><th>Mean</th><th>Standard Deviation</th></tr>
<tr><td>Z-Average (nm)</td><td>76.11</td><td>4.176</td></tr>
<tr><td>Polydispersity Index (PI)</td><td>0.2062</td><td>0.01271</td></tr>
<tr><td>Intercept</td><td>0.9712</td><td>0.002015</td></tr>
<tr><td>Fit Error</td><td>0.001254</td><td>8.129E-05</td></tr>
<tr><td>In Range (%)</td><td>97.11</td><td>0.4563</td></tr>
<tr><td>Peak One Mean by Intensity (nm)</td><td>96.28</td><td>6.188</td></tr>
</table>
</td></tr>
</table>

Table S9. Size distribution of PEO-PBCL$_9$ NPs after freeze-thawing at different freezing conditions.

**Freezing condition**	**Size distribution measured by DLS**	
Room-temperature	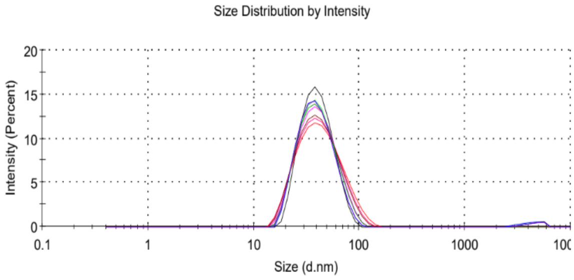	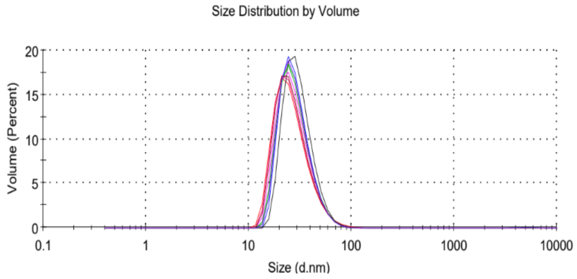
Liquid nitrogen	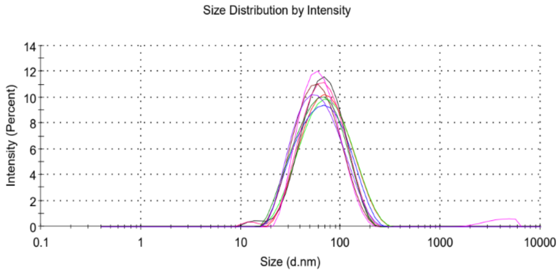	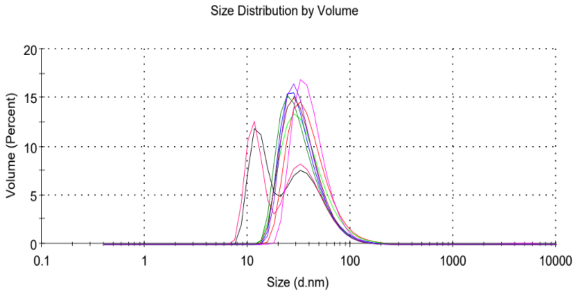
-80°C freezer	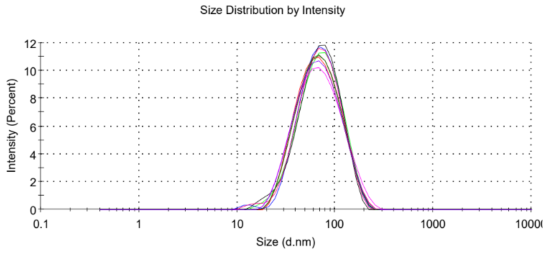	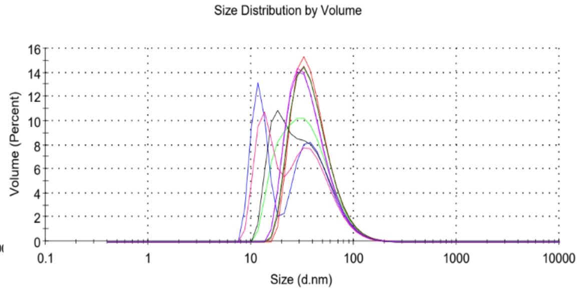
-20°C freezer	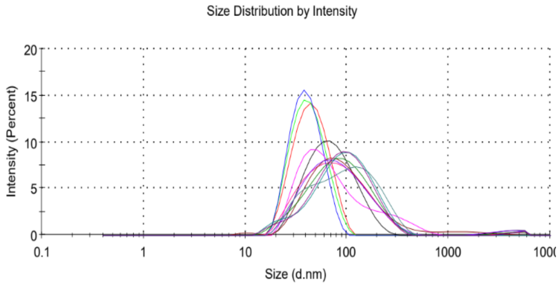	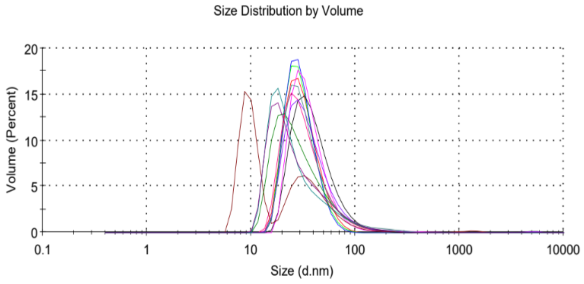

Table S10. Size distribution of PEO-PBCL$_9$ NPs after freeze-thawing with different w/w ratios and Mw of methoxy PEG

	0.25:1	0.5:1	1:1	2:1	4:1

Methoxy-PEG 2000

0.25:1

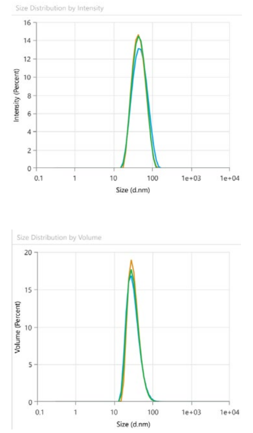

Name	Mean	Standard Deviation
Z-Average (nm)	42.78	0.7118
Polydispersity Index (PI)	0.1374	0.01698
Intercept	0.9556	0.002037
Fit Error	0.0004693	1.521E-05
In Range (%)	97.43	0.3093
Peak One Mean by Intensity (nm)	49.29	1.956

0.5:1

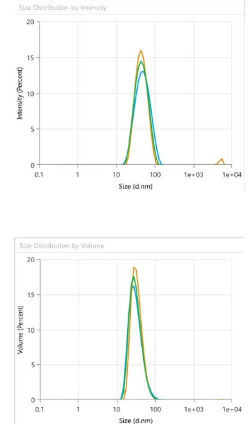

Name	Mean	Standard Deviation
Z-Average (nm)	43.34	0.6263
Polydispersity Index (PI)	0.1713	0.0253
Intercept	0.9577	0.0004742
Fit Error	0.0007061	4.641E-05
In Range (%)	96.44	1.505
Peak One Mean by Intensity (nm)	48.85	2.932
Peak Two Mean by Intensity (nm)	5103	-

1:1

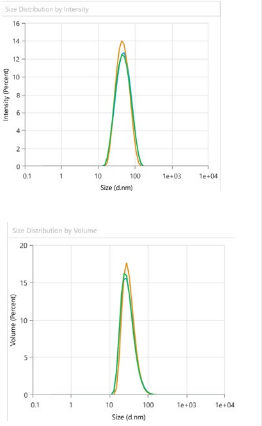

Name	Mean	Standard Deviation
Z-Average (nm)	43.41	0.3747
Polydispersity Index (PI)	0.1571	0.0055
Intercept	0.9597	0.0005549
Fit Error	0.0005964	0.0001382
In Range (%)	97.16	0.3127
Peak One Mean by Intensity (nm)	51.54	1.76

2:1

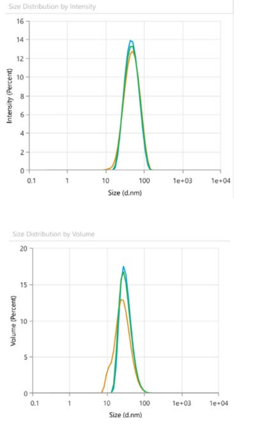

Name	Mean	Standard Deviation
Z-Average (nm)	43.65	0.4195
Polydispersity Index (PI)	0.1606	0.0057
Intercept	0.9608	0.001665
Fit Error	0.00077	7.045E-05
In Range (%)	97.39	0.4495
Peak One Mean by Intensity (nm)	51.7	0.6052

4:1

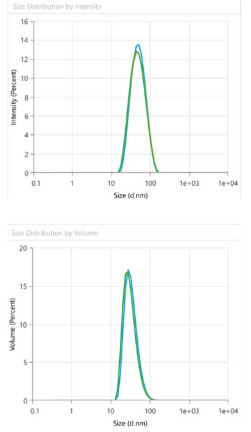

Name	Mean	Standard Deviation
Z-Average (nm)	43.83	0.9735
Polydispersity Index (PI)	0.1627	0.006118
Intercept	0.9644	0.001215
Fit Error	0.0009553	0.000164
In Range (%)	97.48	0.3866
Peak One Mean by Intensity (nm)	52.51	1.018

Methoxy-PEG 5000

0.25:1

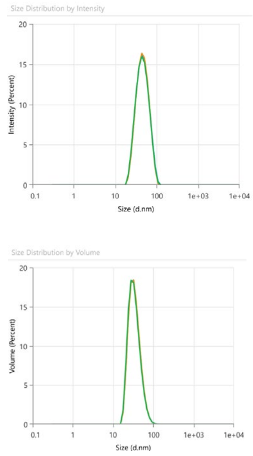

Name	Mean	Standard Deviation
Z-Average (nm)	42.56	0.4623
Polydispersity Index (PI)	0.0998	0.002992
Intercept	0.9621	0.001532
Fit Error	0.0005328	0.0001237
In Range (%)	97.47	0.3229
Peak One Mean by Intensity (nm)	47.77	0.3486

0.5:1

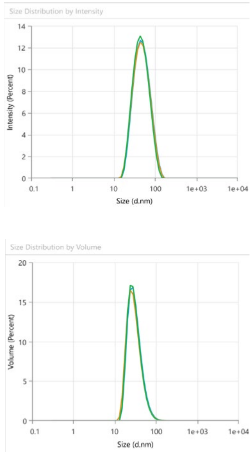

Name	Mean	Standard Deviation
Z-Average (nm)	42.55	0.7085
Polydispersity Index (PI)	0.1575	0.003951
Intercept	0.9646	0.001235
Fit Error	0.0005967	4.826E-05
In Range (%)	97.42	0.402
Peak One Mean by Intensity (nm)	50.9	1.412

1:1

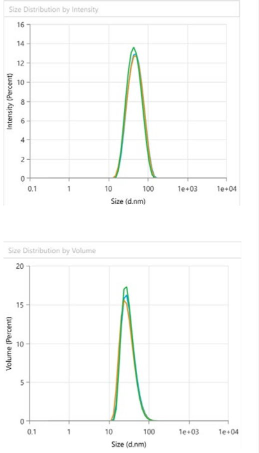

Name	Mean	Standard Deviation
Z-Average (nm)	43	0.6909
Polydispersity Index (PI)	0.1573	0.01197
Intercept	0.9636	0.001593
Fit Error	0.0005736	0.000109
In Range (%)	97.66	0.3972
Peak One Mean by Intensity (nm)	50.63	1.922

2:1

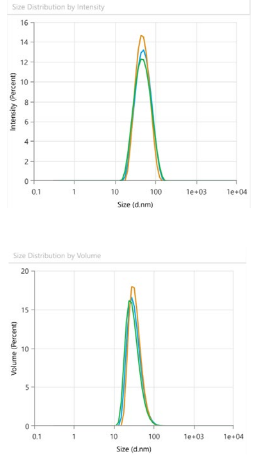

Name	Mean	Standard Deviation
Z-Average (nm)	43.96	0.7233
Polydispersity Index (PI)	0.1628	0.005617
Intercept	0.9661	0.00261
Fit Error	0.0007287	0.0001168
In Range (%)	97.49	0.1882
Peak One Mean by Intensity (nm)	52.49	1.474

4:1

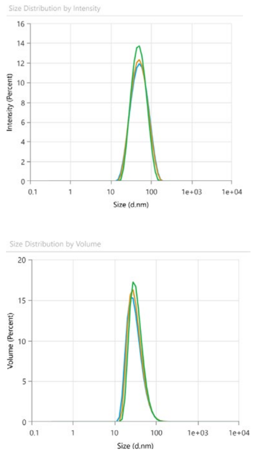

Name	Mean	Standard Deviation
Z-Average (nm)	44.84	0.7454
Polydispersity Index (PI)	0.1891	0.007745
Intercept	0.9756	0.002491
Fit Error	0.001391	7.98E-05
In Range (%)	97.27	0.2817
Peak One Mean by Intensity (nm)	55.25	1.601

No PEG added

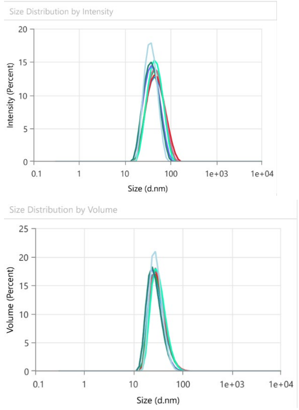

Name	Mean	Standard Deviation
Z-Average (nm)	40.85	3.641
Polydispersity Index (PI)	0.1579	0.01589
Intercept	0.9623	0.01058
Fit Error	0.0005548	0.000138
In Range (%)	96.94	1.124
Peak One Mean by Intensity (nm)	46.62	5.296

Table S11. Size distribution of PEO-PBCL$_9$ NPs after freeze-thawing with different 2:1 w/w of PEG to polymer and 13.25:1 of sucrose to polymer

<table>
<tr><th>Cryoprotectant</th><th>Size distribution</th></tr>
<tr><td>No cryoprotectant before freeze-drying</td><td>
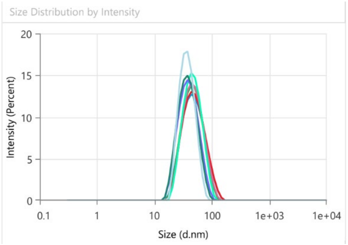

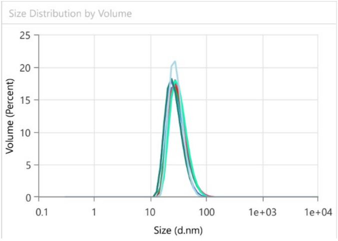

<table>
<tr><th>Name</th><th>Mean</th><th>Standard Deviation</th></tr>
<tr><td>Z-Average (nm)</td><td>40.85</td><td>3.641</td></tr>
<tr><td>Polydispersity Index (PI)</td><td>0.1579</td><td>0.01589</td></tr>
<tr><td>Intercept</td><td>0.9623</td><td>0.01058</td></tr>
<tr><td>Fit Error</td><td>0.0005548</td><td>0.000138</td></tr>
<tr><td>In Range (%)</td><td>96.94</td><td>1.124</td></tr>
<tr><td>Peak One Mean by Intensity (nm)</td><td>46.62</td><td>5.296</td></tr>
</table>
</td></tr>
<tr><td>No cryoprotectant After freeze - drying</td><td>
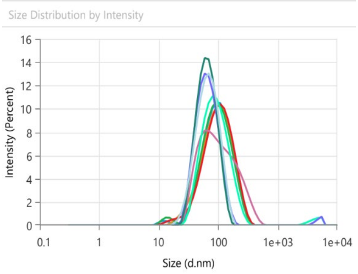

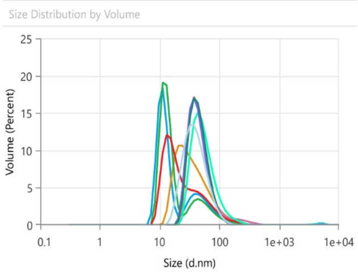

<table>
<tr><th>Name</th><th>Mean</th><th>Standard Deviation</th></tr>
<tr><td>Z-Average (nm)</td><td>73.72</td><td>9.889</td></tr>
<tr><td>Polydispersity Index (PI)</td><td>0.2267</td><td>0.03818</td></tr>
<tr><td>Intercept</td><td>0.9654</td><td>0.004857</td></tr>
<tr><td>Fit Error</td><td>0.001618</td><td>0.0005823</td></tr>
<tr><td>In Range (%)</td><td>96.43</td><td>1.388</td></tr>
<tr><td>Peak One Mean by Intensity (nm)</td><td>96.02</td><td>20.61</td></tr>
<tr><td>Peak Two Mean by Intensity (nm)</td><td>2311</td><td>2662</td></tr>
</table>
</td></tr>
<tr><td>PEG 3350</td><td>
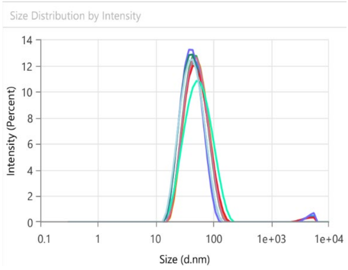

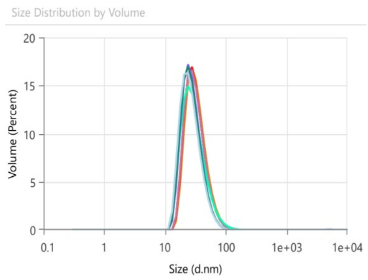

<table>
<tr><th>Name</th><th>Mean</th><th>Standard Deviation</th></tr>
<tr><td>Z-Average (nm)</td><td>43.58</td><td>3.709</td></tr>
<tr><td>Polydispersity Index (PI)</td><td>0.1869</td><td>0.01319</td></tr>
<tr><td>Intercept</td><td>0.9627</td><td>0.004007</td></tr>
<tr><td>Fit Error</td><td>0.000706</td><td>0.0001085</td></tr>
<tr><td>In Range (%)</td><td>97.18</td><td>0.3943</td></tr>
<tr><td>Peak One Mean by Intensity (nm)</td><td>52.05</td><td>5.453</td></tr>
<tr><td>Peak Two Mean by Intensity (nm)</td><td>4470</td><td>230.2</td></tr>
</table>
</td></tr>
<tr><td>Methoxy-PEG 2000</td><td>
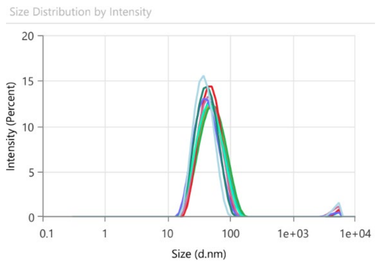

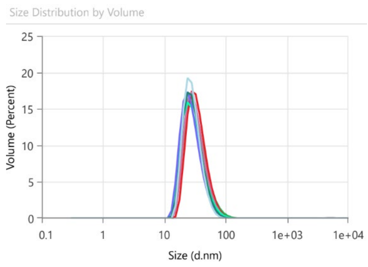

<table>
<tr><th>Name</th><th>Mean</th><th>Standard Deviation</th></tr>
<tr><td>Z-Average (nm)</td><td>43.61</td><td>3.6</td></tr>
<tr><td>Polydispersity Index (PI)</td><td>0.1978</td><td>0.01952</td></tr>
<tr><td>Intercept</td><td>0.9614</td><td>0.007686</td></tr>
<tr><td>Fit Error</td><td>0.0007565</td><td>0.0001466</td></tr>
<tr><td>In Range (%)</td><td>97.17</td><td>0.7786</td></tr>
<tr><td>Peak One Mean by Intensity (nm)</td><td>49.93</td><td>5.881</td></tr>
<tr><td>Peak Two Mean by Intensity (nm)</td><td>4852</td><td>205.8</td></tr>
</table>
</td></tr>
<tr><td>Methoxy-PEG 5000</td><td>
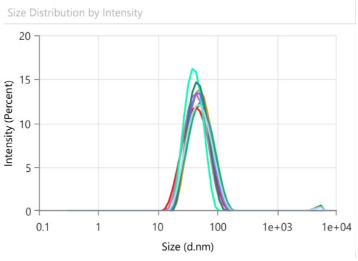

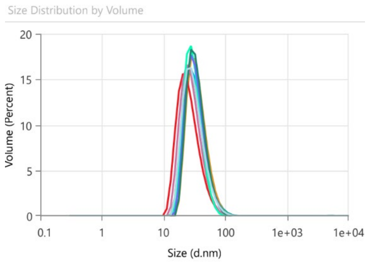

<table>
<tr><th>Name</th><th>Mean</th><th>Standard Deviation</th></tr>
<tr><td>Z-Average (nm)</td><td>43.91</td><td>3.435</td></tr>
<tr><td>Polydispersity Index (PI)</td><td>0.1868</td><td>0.01115</td></tr>
<tr><td>Intercept</td><td>0.9665</td><td>0.002933</td></tr>
<tr><td>Fit Error</td><td>0.0008356</td><td>0.0001291</td></tr>
<tr><td>In Range (%)</td><td>97.43</td><td>0.4506</td></tr>
<tr><td>Peak One Mean by Intensity (nm)</td><td>51.38</td><td>5.346</td></tr>
<tr><td>Peak Two Mean by Intensity (nm)</td><td>4832</td><td>61</td></tr>
</table>
</td></tr>
<tr><td>Sucrose</td><td>
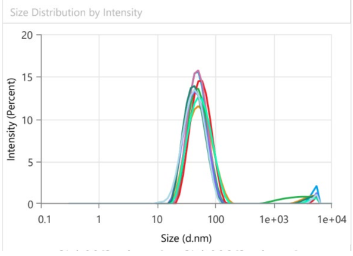

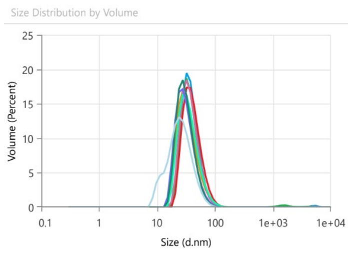

<table>
<tr><th>Name</th><th>Mean</th><th>Standard Deviation</th></tr>
<tr><td>Z-Average (nm)</td><td>48.91</td><td>4.542</td></tr>
<tr><td>Polydispersity Index (PI)</td><td>0.2397</td><td>0.02554</td></tr>
<tr><td>Intercept</td><td>0.9723</td><td>0.003317</td></tr>
<tr><td>Fit Error</td><td>0.000999</td><td>0.0003401</td></tr>
<tr><td>In Range (%)</td><td>95.76</td><td>0.9605</td></tr>
<tr><td>Peak One Mean by Intensity (nm)</td><td>52.88</td><td>5.246</td></tr>
<tr><td>Peak Two Mean by Intensity (nm)</td><td>4498</td><td>800.9</td></tr>
</table>
</td></tr>
</table>

Table S12. Size distribution of PEO-PBCL$_{22}$ NPs after freeze-thawing with different w/w of PEG to polymer and 13.25:1 of sucrose to polymer

Cryoprotectant	Size distribution
No cryoprotectant before freeze-drying	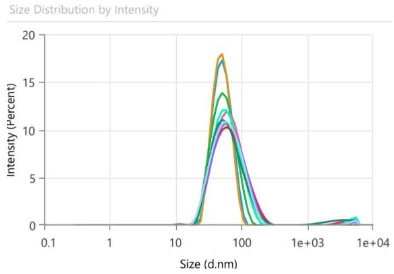 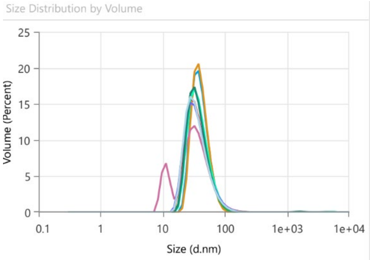
No cryoprotectant After freeze - drying	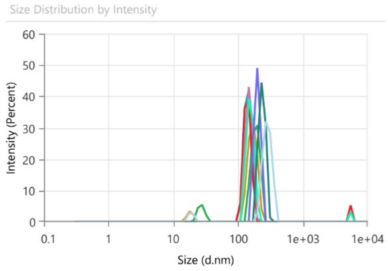 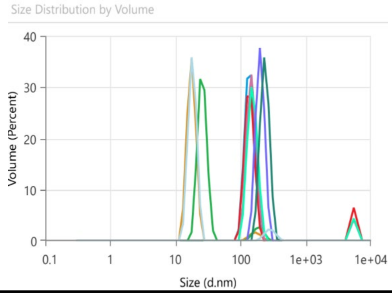
PEG 3350 (w/w 2:1)	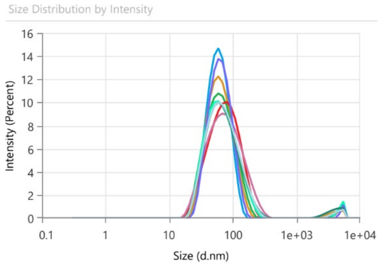 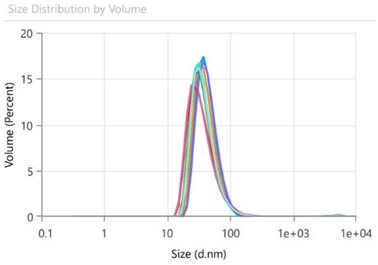
Methoxy-PEG 2000 (w/w 2:1)	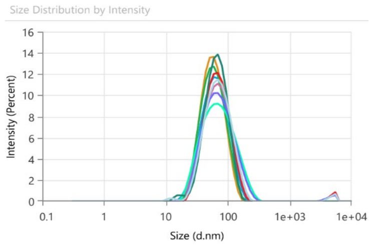 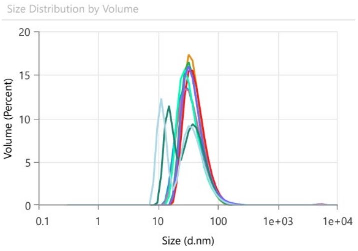
Methoxy-PEG 5000 (w/w 2:1)	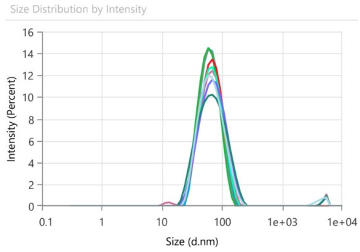 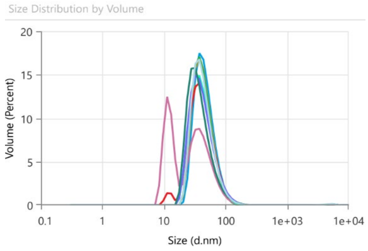
Sucrose	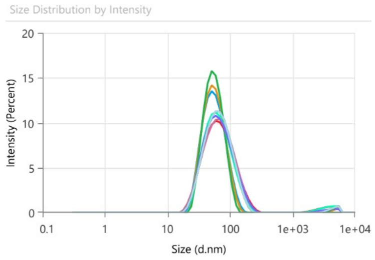 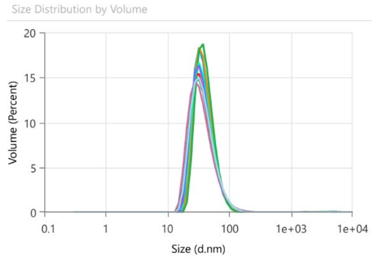

No cryoprotectant before freeze-drying

Name	Mean	Standard Deviation
Z-Average (nm)	54.5	3.67
Polydispersity Index (PI)	0.2013	0.03523
Intercept	0.9655	0.01058
Fit Error	0.0007165	0.0001743
In Range (%)	94.75	1.434
Peak One Mean by Intensity (nm)	63.83	7.984
Peak Two Mean by Intensity (nm)	4409	661.7
Peak Three Mean by Intensity (nm)	11.82	-

No cryoprotectant After freeze - drying

Name	Mean	Standard Deviation
Z-Average (nm)	353.7	126.1
Polydispersity Index (PI)	0.5349	0.05062
Intercept	0.9301	0.01631
Fit Error	0.0105	0.005817
In Range (%)	92.62	1.562
Peak One Mean by Intensity (nm)	183.7	48.69
Peak Two Mean by Intensity (nm)	2744	2983

PEG 3350 (w/w 2:1)

Name	Mean	Standard Deviation
Z-Average (nm)	62.17	2.652
Polydispersity Index (PI)	0.2612	0.0301
Intercept	0.9664	0.00416
Fit Error	0.001066	0.0005094
In Range (%)	94.15	2.319
Peak One Mean by Intensity (nm)	73.97	7.694
Peak Two Mean by Intensity (nm)	4672	465.1

Methoxy-PEG 2000 (w/w 2:1)

Name	Mean	Standard Deviation
Z-Average (nm)	59.58	4.474
Polydispersity Index (PI)	0.2182	0.03334
Intercept	0.9702	0.002501
Fit Error	0.0009855	0.0003884
In Range (%)	96.3	1.274
Peak One Mean by Intensity (nm)	71.56	6.912
Peak Two Mean by Intensity (nm)	3864	2153
Peak Three Mean by Intensity (nm)	12.61	-

Methoxy-PEG 5000 (w/w 2:1)

Name	Mean	Standard Deviation
Z-Average (nm)	62.09	2.968
Polydispersity Index (PI)	0.2215	0.03647
Intercept	0.968	0.005171
Fit Error	0.0009806	0.0003531
In Range (%)	95.13	0.7839
Peak One Mean by Intensity (nm)	70.49	4.303
Peak Two Mean by Intensity (nm)	4194	2065
Peak Three Mean by Intensity (nm)	13.01	-

Sucrose

Name	Mean	Standard Deviation
Z-Average (nm)	57.24	3.963
Polydispersity Index (PI)	0.2119	0.03699
Intercept	0.9695	0.003182
Fit Error	0.0008351	0.0003127
In Range (%)	97.33	0.6495
Peak One Mean by Intensity (nm)	67.65	7.246
Peak Two Mean by Intensity (nm)	4396	500

Table S12. Continues.

<table>
<tr><th>Cryoprotectant</th><th colspan="3">Size distribution</th></tr>
<tr><td>PEG 3350 (w/w 4:1)</td><td>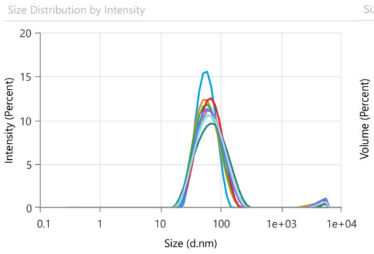
</td><td>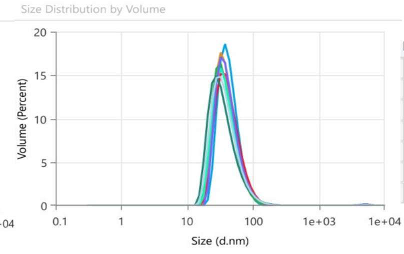
</td><td>
<table>
<tr><th>Name</th><th>Mean</th><th>Standard Deviation</th></tr>
<tr><td>Z-Average (nm)</td><td>60.39</td><td>3.58</td></tr>
<tr><td>Polydispersity Index (PI)</td><td>0.2299</td><td>0.02676</td></tr>
<tr><td>Intercept</td><td>0.9748</td><td>0.00392</td></tr>
<tr><td>Fit Error</td><td>0.001059</td><td>0.0003766</td></tr>
<tr><td>In Range (%)</td><td>95.93</td><td>0.7987</td></tr>
<tr><td>Peak One Mean by Intensity (nm)</td><td>70.99</td><td>6.348</td></tr>
<tr><td>Peak Two Mean by Intensity (nm)</td><td>4635</td><td>374.4</td></tr>
</table></td></tr>
<tr><td>Methoxy-PEG 2000 (w/w 4:1)</td><td>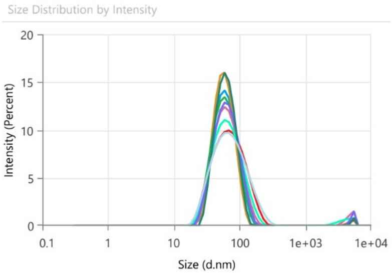
</td><td>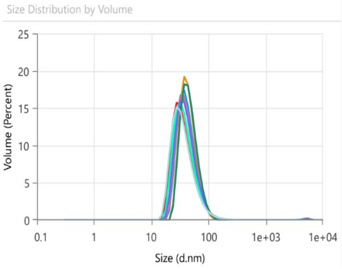
</td><td>
<table>
<tr><th>Name</th><th>Mean</th><th>Standard Deviation</th></tr>
<tr><td>Z-Average (nm)</td><td>59.26</td><td>2.719</td></tr>
<tr><td>Polydispersity Index (PI)</td><td>0.224</td><td>0.04552</td></tr>
<tr><td>Intercept</td><td>0.9735</td><td>0.003801</td></tr>
<tr><td>Fit Error</td><td>0.0009645</td><td>0.0002316</td></tr>
<tr><td>In Range (%)</td><td>95.52</td><td>1.6</td></tr>
<tr><td>Peak One Mean by Intensity (nm)</td><td>68.6</td><td>6.867</td></tr>
<tr><td>Peak Two Mean by Intensity (nm)</td><td>4921</td><td>423.3</td></tr>
</table></td></tr>
<tr><td>Methoxy-PEG 5000 (w/w 4:1)</td><td>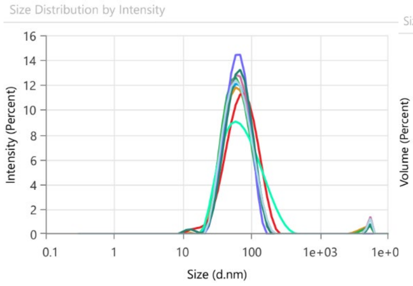
</td><td>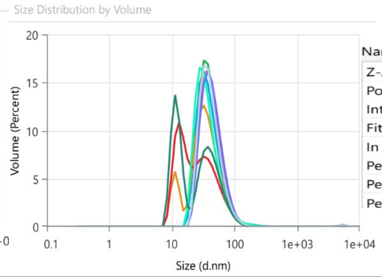
</td><td>
<table>
<tr><th>Name</th><th>Mean</th><th>Standard Deviation</th></tr>
<tr><td>Z-Average (nm)</td><td>61.44</td><td>2.741</td></tr>
<tr><td>Polydispersity Index (PI)</td><td>0.2485</td><td>0.03871</td></tr>
<tr><td>Intercept</td><td>0.972</td><td>0.005675</td></tr>
<tr><td>Fit Error</td><td>0.001053</td><td>0.0002633</td></tr>
<tr><td>In Range (%)</td><td>94.63</td><td>2.464</td></tr>
<tr><td>Peak One Mean by Intensity (nm)</td><td>71.58</td><td>6.965</td></tr>
<tr><td>Peak Two Mean by Intensity (nm)</td><td>3979</td><td>2240</td></tr>
<tr><td>Peak Three Mean by Intensity (nm)</td><td>2653</td><td>3735</td></tr>
</table></td></tr>
</table>
